# Parameter subset reduction for patient-specific modelling of arrhythmogenic cardiomyopathy-related mutation carriers in the CircAdapt model

**DOI:** 10.1098/rsta.2019.0347

**Published:** 2020-05-25

**Authors:** Nick van Osta, Aurore Lyon, Feddo Kirkels, Tijmen Koopsen, Tim van Loon, Maarten J. Cramer, Arco J. Teske, Tammo Delhaas, Wouter Huberts, Joost Lumens

**Affiliations:** 1 Department of Biomedical Engineering, Maastricht University CARIM School for Cardiovascular Diseases, Maastricht, Limburg, The Netherlands; 2 Department of Cardiology, University Medical Center Utrecht, Utrecht, Utrecht, The Netherlands

**Keywords:** Morris screening method, quasi-Monte Carlo, particle swarm optimization, parameter subset reduction, CircAdapt, arrhythmogenic cardiomyopathy

## Abstract

Arrhythmogenic cardiomyopathy (AC) is an inherited cardiac disease, clinically characterized by life-threatening ventricular arrhythmias and progressive cardiac dysfunction. Patient-specific computational models could help understand the disease progression and may help in clinical decision-making. We propose an inverse modelling approach using the CircAdapt model to estimate patient-specific regional abnormalities in tissue properties in AC subjects. However, the number of parameters (*n* = 110) and their complex interactions make personalized parameter estimation challenging. The goal of this study is to develop a framework for parameter reduction and estimation combining Morris screening, quasi-Monte Carlo (qMC) simulations and particle swarm optimization (PSO). This framework identifies the best subset of tissue properties based on clinical measurements allowing patient-specific identification of right ventricular tissue abnormalities. We applied this framework on 15 AC genotype-positive subjects with varying degrees of myocardial disease. Cohort studies have shown that atypical regional right ventricular (RV) deformation patterns reveal an early-stage AC disease. The CircAdapt model of cardiovascular mechanics and haemodynamics has already demonstrated its ability to capture typical deformation patterns of AC subjects. We, therefore, use clinically measured cardiac deformation patterns to estimate model parameters describing myocardial disease substrates underlying these AC-related RV deformation abnormalities. Morris screening reduced the subset to 48 parameters. qMC and PSO further reduced the subset to a final selection of 16 parameters, including regional tissue contractility, passive stiffness, activation delay and wall reference area.

This article is part of the theme issue ‘Uncertainty quantification in cardiac and cardiovascular modelling and simulation’.

## 1. Introduction

Arrhythmogenic cardiomyopathy (AC) is an inherited cardiomyopathy, clinically characterized by the occurrence of ventricular arrhythmias, sudden cardiac death (SCD) and predominantly right ventricular (RV) dysfunction [1,2]. A pathogenic genetic mutation, mostly affecting desmosomal genes, is found in up to 60% of probands. This mutation may result in the fibro-fatty replacement of the myocardium, which can be a substrate for life-threatening arrhythmias and may already occur in an early stage without overt signs of disease using conventional screening tools [3]. To prevent SCD in these apparently healthy AC mutation carriers, early detection of pro-arrhythmic tissue substrates is important.

The diagnosis of AC is based on a set of criteria described in the revised 2010 Task Force Criteria (TFC), with the electrocardiogram (ECG) and cardiac imaging as central elements [4]. By using these conventional screening tools, mutation carriers can be classified into three categories: (i) a concealed stage with no abnormalities, (ii) an electrical stage with electrical abnormalities, but no structural abnormalities, and (iii) a structural stage with both electrical and structural abnormalities. Conventional electrocardiographic and structural imaging methods as described in the 2010 TFC are specific but may lack sensitivity to detect the early-stage AC-related myocardial disease in genotype-positive AC patients and family members [5].

Mast *et al*. [5] found local RV deformation abnormalities in the absence of electrocardiographic and structural 2010 TFC. These abnormal deformation patterns were derived from speckle tracking echocardiography in individuals with a pathogenic Plakophillin-2 (PKP2) or Desmoglein-2 (DSG2) mutation [5]. In a follow-up study by the same group, these deformation abnormalities were related to disease progression from a concealed stage to a stage where subjects had developed electrical or structural abnormalities [6], which suggests that identifying the underlying substrate responsible for these strain abnormalities would help us understand disease progression and ultimately help in arrhythmic risk stratification.

In the initial study by Mast *et al*. [5], generic *in silico* patient simulations were performed using the CircAdapt model. This model is a biophysical model of the human heart and circulation describing the mechanistic link between myocardial tissue properties and regional myocardial strain based on well-established physical and physiological principles [7]. These simulations suggest that regional RV strain abnormalities in AC patients originate from regional contractile dysfunction either or not in combination with myocardial stiffening.

Generic simulations as described above give insight into the disease origin for the generic population but do not contain patient-specific data. Therefore, we hypothesize that cardiac deformation imaging-based personalization of the CircAdapt model not only may reveal the severity of AC-related RV tissue disease but also may help to monitor or even to predict disease progression and therefore may support clinical decision-making.

To use the CircAdapt model for clinical decision support in AC subjects, it is essential to assess which model parameters can be uniquely identified given this model and available data. The CircAdapt model as configured for this study contains 110 parameters. Owing to mechanical interactions within and between the walls of the heart, on the one hand, and between heart and circulation, on the other hand, the model acts highly nonlinear, non-monotone and non-additive. This behaviour, combined with the limited amount of available clinical data, challenges the identifiability of the parameters. Therefore, patient-specific modelling of cardiac deformation characteristics is challenging.

Reducing the dimensionality of the model is necessary for patient-specific modelling. This can be done with a screening method such as Morris screening [8]. It is based on a one-at-a-time method, where the effect of individual parameter changes on the model output is evaluated. The method is designed to account not only for their individual effect but also interactions. It has been demonstrated to be a proxy for the variance-based total sensitivity index of a parameter [9]. Therefore, it is a suitable method to reduce the dimensionality of the model.

Owing to the complexity of the model, Morris screening does not result in a sufficiently reduced parameter subset. By performing actual runs of patient-specific parameter estimation using quasi-random Monte Carlo simulations, the quality of estimation using different parameter subsets can be assessed and compared. Using the diaphony [10] obtained from quasi-random Monte Carlo simulations, the relative importance of parameters can be determined, which guides the subset reduction. Particle swarm optimization (PSO) [11,12] can be used for parameter estimation. Because the subset reduction is based on the size of parameter input space, results are biased. By comparing the estimations following from PSO, the subset reductions are validated independent of input space size.

The goal of this study is to introduce a parameter reduction and estimation framework allowing personalization of models with many parameters. Though this approach is more widely applicable, we focus on the identification of the best subset of model parameters essential to accurately simulate patient-specific RV deformation using the CircAdapt model. This framework reduces the parameter subset in two steps based on importance and identifiability. First, the Morris screening method (MSM) was applied to exclude the unimportant parameters given the choice of the model output and the model structure. Second, a quasi-Monte Carlo (qMC) fitting algorithm was used on the reduced parameter subset to determine the parameters that are identifiable by comparing simulate RV deformation curves to measured RV deformation in AC-related mutation carriers. Parameter reduction was done based on the importance and identifiability of the parameters and validated using PSO.

## 2. Methods

### (a) Patient cohort

A total of 15 AC subjects (6 males and 9 females, age 21.0 ± 15.2 years) were included in this study. The subjects were selected from a larger cohort from the University Medical Center Utrecht, which has been previously described in more detail by Mast *et al*. [5]. The study was approved by the local institutional ethics review board. The selected cohort was guaranteed to contain subjects with PKP2 and DSG2 mutations and to cover a wide range of AC disease severity according to the 2010 TFC [4]. Six of the included subjects were in a concealed stage, four showed electrical criteria and five also showed structural criteria.

**Figure 1.**
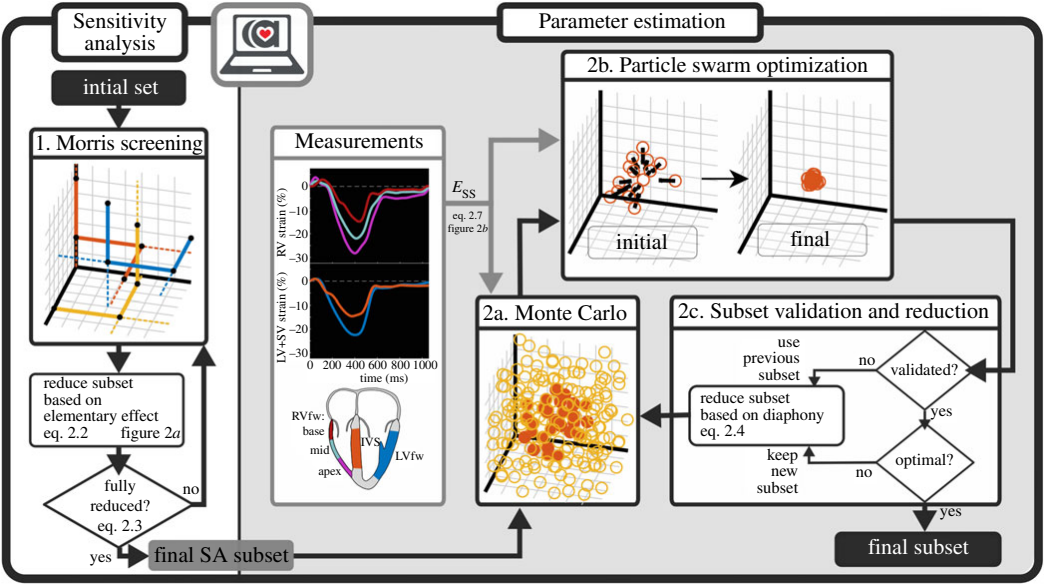
Visualization of the two-step approach. In the first step, sensitivity analysis (SA) is performed using the MSM, which is applied iteratively on the CircAdapt model. Based on the elementary effect, a parameter selection is done. The final SA subset is used in qMC. Using the best estimations for each patient from qMC as a starting point, PSO is applied. Based on PSO results, the previous reduction is validated. Using the diaphony, the subset is further reduced. (Online version in colour.)

The echocardiographic protocol has been described in more detail elsewhere [13]. In brief, all data were obtained on a Vivid 7 or a Vivid E9 ultrasound machine (GE Healthcare, Horten, Norway) using a broadband M3S transducer. All echocardiographic studies were analysed for structural abnormalities fulfilling the 2010 TFC [4]. To quantify myocardial deformation, the conventional apical 2-, 3- and 4-chamber views and the RV-focused 4-chamber view were stored for offline left ventricular (LV) and RV deformation analysis by a single operator, blinded for clinical data. Two-dimensional speckle tracking was performed using EchoPAC v. 202 (GE Healthcare, Horten, Norway), in order to obtain longitudinal strain curves of the LV free wall (LVfw), inter-ventricular septum (IVS) and the apical, mid and basal segment of the RV free wall (RVfw). LV strain curves were acquired according to the standardized 18-segment model [14]. For IVS deformation, the six regional septal strain curves were averaged. For the LVfw deformation, the remaining 12 curves were averaged. The ‘Measurements’ panel of figure 1 shows an example of measured deformation, whereby decreasing longitudinal strain corresponds to shortening of the tissue and increasing strain to lengthening.

### (b) Cardiac mechanics model: the CircAdapt model

The CircAdapt model is a lumped parameter model simulating haemodynamics and wall mechanics of the heart and circulation. The one-fibre model links global haemodynamics to local wall tension by assuming spherical walls [15]. The simplified ‘TriSeg’ ventricular geometry couples the LVfw and the RVfw via the IVS allowing inter-ventricular interactions [16]. The ‘MultiPatch’ module [7] subdivides the RVfw into three segments with equal wall tension, allowing heterogeneity in tissue properties within the RVfw. No abnormal LVfw and IVS strains were found in the cohort. Therefore, tissue properties in the IVS and the LVfw were assumed homogeneous, and, thus, the tissue was modelled as a single segment representing the mechanics of the entire wall. Phenomenological models of active and passive myofibre stress generation were used to describe tissue mechanics of each wall segment [7]. A more detailed description is provided in the electronic supplementary material.

A total of 110 CircAdapt parameters were identified and included in the analysis. This set consists of a group of 60 ventricular parameters (i.e. 12 parameters for each of the three RVfw segments, the IVS and the LVfw) and a group of 50 parameters belonging to the other non-ventricular modules of CircAdapt, such as pulmonary and systemic circulations, valves and atria. All parameters are presented, along with their value range, in electronic supplementary material, table S1. The parameter input space was based on reference values as used in the previous work [7]. Because AC is mostly affecting myocardial tissue properties in the RV basal region in this cohort [5], a wide range was assigned to parameters describing tissue properties in both the RV midventricular and basal segment. By contrast, a smaller range was assigned to all other parameters to account for inter-subject variability.

Engineering strain of the sarcomere was extracted from the model and compared with the measured strain. In the measured strain, the reference length was taken as the length at the onset of the QRS complex, which was obtained using an ECG. To calculate engineering strain in the model, the reference length was defined as the length at the estimated onset of the QRS complex as done in previous studies [17].

### (c) Two-Step parameter subset reduction approach

Figure 1 shows an overview of the methodology used for parameter subset reduction. We applied a two-step approach composed of screening in the first step and parameter estimation and reduction in the second. This framework works with normalized input and uses the output of the model for subset reduction. Therefore, the framework is invariant of the CircAdapt model and could be applied to various other models.

In the first step, we performed MSM iteratively to screen the input space and eliminate unidentifiable parameters given the chosen input space size of the model, as well as the chosen output measures. In the second step, we performed qMC simulations to identify preferred areas within the sample input space (i.e. the best realizations) where the modelled strain was close to the measured strain. These areas indicated the identifiability of the parameters relative to the input space, where unidentifiable parameters are expected to be distributed uniformly and identifiable parameters are expected to have a preferred area. Using these results, the parameter subset was reduced by omitting non-identifiable parameters. We applied PSO for accurate parameter estimation to validate the reduced subset whether the reduction was not induced by the choice of the input space size. This is done by comparing the average goodness of fit of the cohort, obtained with different parameter subsets. MSM and PSO were repeated until further reduction was not beneficial.

#### (i) Step 1: sensitivity analysis using MSM

**Figure 2.**
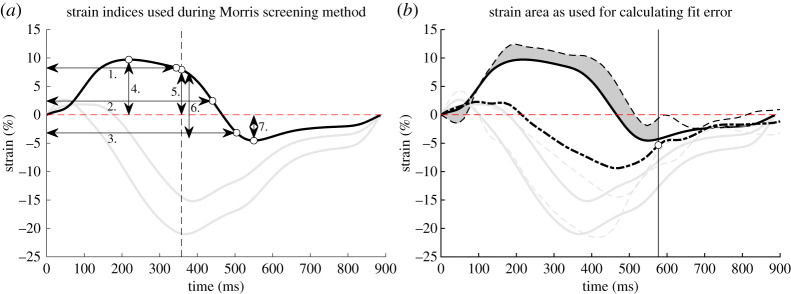
(*a*) Strain indices for one strain curve as used in the MSM. The same indices are included for the three RVfw segments, as well the LVfw and IVS segment. The indices are time to 10%, 50% and 90% shortening (1,2,3), pre-stretch (4), systolic strain (5), post-systolic strain (6) and peak strain (7). The verticle dashed line indicates closure of the pulmonary valve. (*b*) An area is used to calculate the fit error. The area is defined from onset of QRS to 50% relaxation (the white dot with the vertical line) of the global RVfw strain (thick dashed line) and is calculated for all three RVfw segments, as well the LVfw and IVS segment. For the actual fit error, the area for all three RVfw segments, LVfw segment and IVS segment is included. (Online version in colour.)

MSM was used to identify parameters that are unimportant to the model output, irrespective of the input–output space response with respect to linearity, monotonicity and additivity [8]. A subset of relevant parameters was identified for each model output. As the model output, we selected the metrics as shown in figure 2*a*, which together describe the morphology of regional strain patterns. These metrics are time to 10%, 50% and 90% total shortening (1,2,3), pre-stretch (4), systolic strain (5), post-systolic strain (6), and peak strain (7).

The input space $\Omega =R^{D}$ was spanned by all model parameters (initially, *D* = 110), for which their domains were linearly normalized to a range from 0 to 1 resulting in dimensionless parameters. Upper and lower bounds are shown in electronic supplementary material, table S1. The input space was discretized into a *z*-level grid (*z* = 8) with points $x_{i}\in \{0,(1/z-1),\ldots ,1\}$ and *i* ∈ {1, 2, …, *D*}. Each trajectory started at a randomly chosen point on the grid and changed parameter values in a random order one-at-a-time with step size Δ=(1/2)z/z−1, such that all points on the grid were equally likely to be included [8]. CircAdapt was used to calculate each output *Y_j_* for each input point ***x***.

The elementary effect *E* of parameter *i* on output *j* in trajectory *r* is given by the following equation:

2.1 $$E_{ij}(r)=\frac{Y_{j}(x+e_{i}\Delta )-Y_{j}(x)}{\Delta }$$

with ***x*** any point in the *z*-level grid, and ***e****_i_* is the unit vector in the direction *i*. To quantify the total elementary effect, the absolute average $\mu_{ij}^{*}$ of all trajectories *N_r_* [9], with *r* ∈ {1, 2, …, *N_r_*} is calculated as follows:

2.2 $$\mu_{ij}^{*}=\frac{1}{N_{r}}\sum_{r=1}^{N_{r}}E_{ij}(r).$$

This elementary effect sensitivity measure $\mu_{ij}^{*}$ is considered a proxy of the variance-based total sensitivity index [9]. Consequentially, the unimportant parameters could be considered insensitive to the model output and therefore omitted from the parameter subset. A parameter input *X_i_* was considered to be unimportant if it is unimportant for all output *Y_j_*, i.e.

2.3 $$\mu_{ij}^{*}<\frac{1}{D}\sum_{k=1}^{D}\mu_{kj}^{*}\forall j.$$

We aimed for 1000 successfully completed trajectories to represent the entire input space. Convergence check was done using a leave-one-out method on all trajectories, i.e. when omitting one trajectory, the total set of sensitive parameters should not change. A new subset including all relevant parameters was defined. MSM was repeated until no parameters could be omitted to reduce the input space as much as possible, which resulted in the final sensitivity analysis (SA) subset (figure 1).

#### (ii) Step 2a: parameter estimation using qMC

The simulations obtained with qMC were used to identify preferred areas in the input space which best described the cohort data and thereby identified the parameters that have the most influence on the fits within the chosen input space size. In this step, the model output was compared to the measured strain. Within the input space, one million quasi-random simulations were performed for which the input samples were generated using the Sobol low-discrepancy sequence [18].

For each patient, the fit error as described below was calculated for each simulation. Because simulations may become numerically unstable in case of non-physiological parameter combinations, the error for these crashed simulations was set to infinite. For each parameter subset for each patient, a distribution plot containing the best 100 simulations was made for visualization of the input space for these simulations. To quantify parameter preference, the diaphony *d_i_* [10] of the best *N*_b_ = 100 simulations for each patient was calculated for each parameter *i* as follows:

2.4 $$d_{i}=\left|\frac{1}{N_{b}}\sum_{N_{b}}e^{i2\pi x_{i}}\right|.$$

Thereby, the diaphony was used as a measure for homogeneity, with *d_i_* = 0 for a completely homogeneously distributed sample set and *d_i_* = 1 when all parameters had the same value.

#### (iii) Step 2b: parameter estimation using PSO

Unless qMC runs with a very large number of samples, the qMC estimations do not find an accurate fit due to the large input space and can only be used to find trends in parameter preference. For validation of the parameter subset reduction based on qMC, the global minimum of each subset should be found and compared. For this purpose, PSO was applied [11,12].

PSO is an evolutionary algorithm, where a population of candidate solutions move through the input space driven by their own history and the history of the population. For PSO, the simulations with least fit error from qMC were used as starting points to improve the initial solution guess. Initially, the speed was set to zero. During each iteration, a dimensionless velocity ***v****_t_*_,*k*_ of particle *k* was updated depending on its previous velocity and the distance to the local and global minima, by using

2.5 $$v_{t+1,k}=w\cdot v_{t,k}+c_{1}\cdot Z_{1}\cdot (x_{t,k}-x_{p_{t,k}})+c_{2}\cdot Z_{2}\cdot (x_{t,k}-x_{t,g})$$

with ***x****_t_*_,*k*_ is the dimensionless location of particle *k* at time *t*, $x_{p_{t,k}}$ its local optimum, ***x****_t_*_,*g*_ is the swarms optimum, *w* is the inertia damping factor, *c*_1_ and *c*_2_ are the inertia constants to change the velocity towards the local and global optimum and ${\text{Z}}_{i}$ is a vector with random numbers following the standard uniform distribution Z∈U(0,1). The same settings were chosen as used by Eberhart & Shi [19], i.e. a population size of 30, and using dimensionless constant values which met Clerc's constriction method to enforce convergence [20], i.e. *w* = 0.729 and *c*_1_ = *c*_2_ = 1.49445.

The fit error was set to be infinite when the particle was outside the defined parameter space (electronic supplementary material, table S1) to ensure physiological values. We assumed a particle *k* converged when the dimensionless ‘energy’ was below 0.1 for all particle. The energy of a particle was defined as follows:

2.6 $$E_{k}=v_{k}^{2}+{\left(x_{t,k}-x_{p_{k}}\right)}^{2}+{\left(x_{t,k}-x_{g}\right)}^{2}.$$

Simulations were stopped when all particles converged, or when the limit of 1000 iterations was reached.

#### (iv) Step 2c: subset validation and reduction

Using the fit error resulting from PSO, the previous subset reduction was validated. If the summed squared error (SSE) in the reduced subset was similar to that in the larger subset, the reduced subset was accepted and the further reduction was applied. If not, another reduction of the previous subset was performed. The subset was considered optimal when all parameters were identifiable, and, thus, no further reduction could be made.

Reduction of the subset was based on the diaphony, which was calculated for each parameter in each patient separately. For each parameter, the maximum diaphony of the subjects is used. The parameters with the lowest diaphony were omitted in the new subset.

### (d) Fit error

In MSM, no patient data was included and strain indices were identified to find the identifiability of model parameters to these indices. In qMC and PSO, however, patient data was included. As discussed before, it is unknown which combination of strain indices fully captures the myocardial strain patterns in all segments. To avoid that the parameter reduction is influenced by the choice of the strain indices, the whole strain curve of all segments was used to compute the fitting error. To reduce the effect of drift in the measurement, only strain from the onset of the QRS complex up to 50% of relaxation of the global RV strain was included as shown in figure 2.

The fit error as used in qMC and PSO is composed of the squared error of each segment, $A_{\mathrm{set}}^{2}$, which uses the difference in modelled strain *ε*_model,seg_ and measured strain *ε*_meas,seg_ and is defined by the following equation:

2.7 $$A_{\mathrm{seg}}^{2}=\int_{t_{\mathrm{onset}\;\mathrm{QRS}}}^{t_{50\;\mathrm{relax}}}{\left(\epsilon_{\mathrm{model},\mathrm{seg}}-\epsilon_{\mathrm{meas},\mathrm{seg}}\right)}^{2}dt.$$

From preliminary studies, it is known that if modelled and measured RV strain match, modelled LVfw and IVS strain curves do not necessarily have to match the measured data. Therefore, we not only used all three RVfw segments but also constrained the strain of IVS and LVfw to fit.

The fit error also contains the error in cycle time. The cycle time *T* (s) was included in the input space to perform one qMC for all AC datasets. Therefore, the squared difference between modelled and measured cycle time *T* (s) was included in the fit error (*E*_SS_), which is defined as follows:

2.8 $$E_{\mathrm{SS}}=\alpha (A_{\mathrm{RV}\;\mathrm{apex}}^{2}+A_{\mathrm{RV}\;\mathrm{mid}}^{2}+A_{\mathrm{RV}\;\mathrm{base}}^{2}+A_{\mathrm{IVS}}^{2}+A_{\mathrm{LVfw}}^{2})+\beta \cdot {\left(T_{\mathrm{model}}-T_{\mathrm{meas}}\right)}^{2}$$

with *α* = 1 s^−1^ and *β* = 0.1 s^−2^.

### (e) Numerical implementation

To reduce the computational cost, a C++ version of the CircAdapt model as in Walmsley *et al*. [7] was used. Equations were linearized using the Newton–Raphson method and were time-integrated using the Adams–Bashford method with a variable timestep Δ*t* with max(Δ*t*) = 2 ms. All other codings, including MSM, qMC and PSO, was done in Matlab 2018a (MathWorks, Natick, MA, USA). Simulations were run on a PC with 16 GB RAM and an Intel i7-8700 (3.2 GHz, 6 cores). All used code is available in the electronic supplementary material.

## 3. Results

### (a) Morris screening method

Out of 3000 trajectories, 1014 were successful in the sense that all simulations converged. Non-converged simulations had numerical instabilities due to parameter value combinations being non-physiological. In the successful trajectories, 48 parameters were relevant for all strain indices. Especially tissue properties of the ventricles were important to describe ventricular strain. Relative parameters were not dependent on one single trajectory, i.e. by leaving one trajectory out, the same parameters remained important.

The second screening step used 2500 trajectories, of which 1064 were successful. In this step, three parameters were unimportant and were omitted. The third step used 2000 trajectories and 1016 were successful. In this step, only one parameter was unimportant. Because the computational cost outweighed the expected parameter reduction, no further screening was done. Remaining parameters in the final subset after screening were all related to LVfw, IVS or RVfw tissue properties except for one parameter related to the left atrium. More details on screening results and important parameters are shown in the electronic supplementary material.

### (b) Subset reduction: qMC and PSO

One million qMC simulations were run multicore with a total computational time of 4 days for each subject. The computational time of PSO that ran single core was approximately 8 h for each subject.

**Figure 3.**
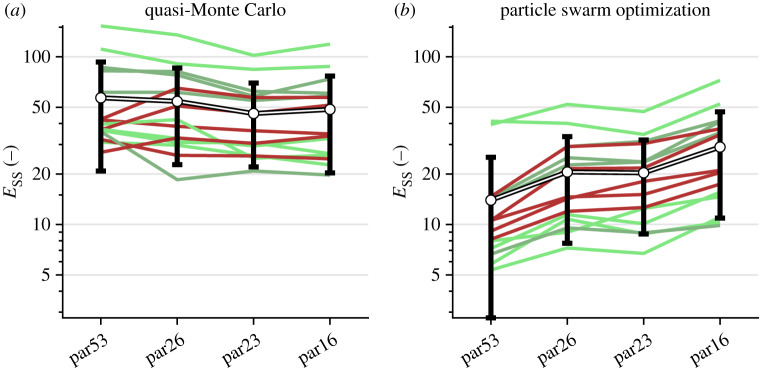
Minimum summed squared error (*E*_ss_) after qMC (*a*) and after PSO (*b*) of the subsets, where parX indicates the number of parameters included. Green lines indicate subjects in the concealed stage, the light and dark red lines indicate subjects in the electrical and structural stage. Black–white lines show the average summed squared error of all subjects including standard deviation. (Online version in colour.)

For the initial qMC subset, we added the five parameters in the RV wall to the 48 parameters remaining from MSM to ensure that all three segments had the same properties. This resulted in a total of 53 parameters (par53). For par53, the success rate of simulations was 97%. Other simulations had numerical instabilities due to similar reasons as in MSM. The minimum diaphony was 0.0674 and the maximum diaphony was 0.9722. The SSE of the best estimation of qMC and PSO are shown in figure 3. As expected, all SSE from PSO were lower than SSE from qMC.

The first reduction omitted the remaining atrial parameter, the relaxation time constant of the LVfw and IVS, the shortening velocity in the LVfw, wall volume of the three RVfw segments and a passive stiffness-related parameter in the three RVfw patches, resulting in 40 parameters (par40). The success rate was 98%, and the best 100 realizations had a minimum and maximum diaphony of 0.12 and 0.97, respectively.

The second reduction omitted LVfw and IVS volume, the contraction time constant in the three RVfw patches, shortening velocity in the three RVfw patches and mean arterial pressure, resulting in 31 parameters (par31). The success rate was 98%. The minimum diaphony was 0.080 and the maximum diaphony was 0.97.

The third reduction omitted the contraction time constant in the LV, and the zero-stress sarcomere length in IVS and the three RVfw patches, resulting in 26 parameters (par26). The success rate was 98%. The minimum diaphony was 0.10 and the maximum diaphony was 0.97.

In the fourth reduction, the linear stiffness component of the three RVfw patches was omitted, resulting in 23 parameters (par23). The success rate was 97%. The minimum and maximum diaphony were 0.13 and 0.97, respectively.

In a fifth step, all LVfw and IVS parameters were removed, resulting in 16 parameters (par16). For this set, the success rate was 96% with the minimum and maximum diaphony 0.098 and 0.97, respectively.

The minimum error in qMC and the error of the global minimum of the PSO for the subsets par53, par26, par23 and par16 are shown in figure 3. By decreasing the parameter subset size, the average error of qMC decreased for all subsets. By applying PSO, the mean *E*_SS_ decreased for all subsets compared to the *E*_SS_ after qMC. Decreasing the number of parameters increased the mean *E*_SS_ from 14 in par53 to 21 in par23 after applying PSO. Removing LVfw parameters from par23 to par16 resulted in the largest increase in SSE from 21 in par23 to 29 in par16 after applying PSO.

### (c) Fits

**Figure 4.**
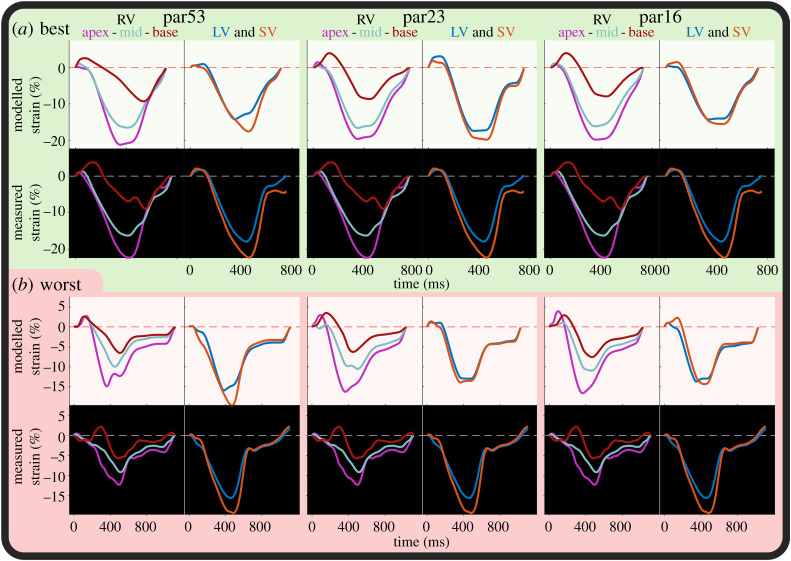
Example fits from PSO of par53 (left), par23 (middle) and par16 (right) of the best fit ((*a*); subject 12) and worst fit ((*b*); subject 14) of subjects with abnormal strain. For subject 12, the summed squared error was 8.2, 13 and 17 for par53, par23 and par16, respectively. For subject 14, the summed squared error was 14, 30 and 37, respectively. (Online version in colour.)

**Figure 5.**
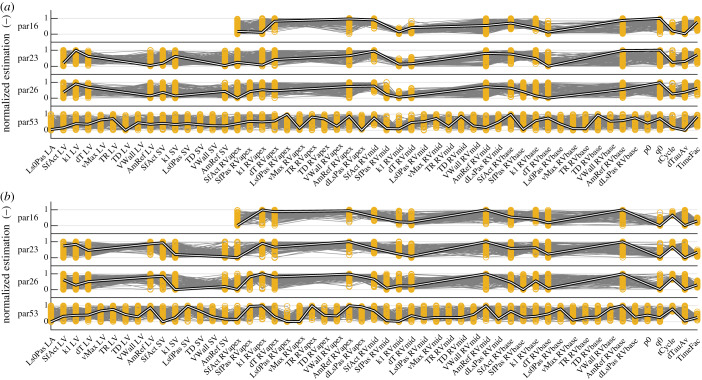
Normalized estimations resulting from qMC and PSO of subject 12 (*a*) and subject 14 (*b*) of the subset with 53, 26, 23 and 16 parameters (par53, par26, par23 and par16, respectively). Included parameters are indicated by the yellow bars. The best 100 simulations from qMC are shown in grey, the global optimum from PSO is shown in the thick black–white line. (Online version in colour.)

An example strain fit for each of the subsets par53, par23 and par16 is shown in figure 4. In par53, par23 and par16, modelled strains are still similar to measured strains despite the increase in fit error. In the par23 and par16, RV strain fit quality is similar. By eyeballing, all parameter subsets are able to capture the strain morphology. The qMC distribution and PSO estimations of these fits are shown in figure 5. Estimated parameter values in par16 are similar to the estimated parameters in the larger subsets.

## 4. Discussion

We successfully designed a new framework to find non-identifiable parameters and to select and estimate a small subset of identifiable parameters from the CircAdapt model to simulate regional RV tissue deformation in AC mutation carriers. This subset could be used to simulate patient-specific RV strain in AC mutation carriers revealing specific regional heterogeneities of RV tissue properties. This selection is done in a two-step approach. First, using MSM, the number of parameters successfully reduced from 110 to 48. Second, using qMC and PSO, the subset was further reduced to 16 parameters, including local contractility, passive stiffness, activation delay and wall reference area of the RVfw, as well as cardiac output, heart rate, AV-delay and relative systole duration (i.e. myofibre twitch duration).

### (a) MSM allows reduction of the parameter subset

It is demonstrated that Morris screening is a successful method to identify unimportant parameters in cardiovascular models. Donders *et al*. found that 16 out of 73 parameters were important in a model for brachial flow and systolic radial pressure using MSM [21]. MSM has also been applied to an LV finite-element model [22] to investigate the effect of model parameters on the local strain. A total of three parameters in 16 segments were included in the SA, but the model contained many more parameters. The authors only included measured time to peak strain, decreasing the complexity of the problem.

In this study, MSM removed over half of all parameters in three screening rounds. The relevance of a parameter depends on its own behaviour but also on the chosen parameter value range. Since simulations in the three screening rounds were not compared to the measurements, we could not validate the chosen range. Therefore, the relevance of some parameters might be under- or overestimated. We presumed that parameters identified by MSM as non-identifiable were not important to model patient-specific strain in AC-related mutation carriers because AC is a myocardial pathology and all ventricular tissue-related parameters were still in the subset. On top of that, in a preliminary one-at-a-time study (results not shown), these parameters did not influence strain significantly compared to parameters related to the pathology. Based on these observations, we concluded that we did not miss out any important parameters in the subset resulting from MSM.

### (b) qMC and PSO allows further reduction based on clinical strain

Further decrease in the number of parameters was done by applying qMC. Validation was done with PSO. Within the smaller subsets par23 and par16, all parameters were identifiable, and therefore, the subset is usable for patient-specific parameter estimation. For these subsets, modelled strain sufficiently resembled the measured strains.

PSO has been previously used for parameter estimation in a study by Mineroff *et al*. [23], who, interestingly, also used the CircAdapt model. They used the CircAdapt model to model volumes and pressures in the cardiac cycle and showed that PSO is a suitable algorithm for patient-specific estimation of parameters in the CircAdapt model. A total of nine parameters were estimated combining cardiac tissue properties and circulation parameters.

Although only a small part of the input space has been explored by qMC, trends in parameter values can be seen already in the largest subset (par53). In the smaller parameter sets (par31, par26, par23 and par16), there were more parameters with a larger diaphony, suggesting that the model converged to a unique point.

### (c) Selected parameters may describe AC disease progression

Parameters included in the final subset (par16) are likely to be related to AC disease progression. As earlier hypothesized, our framework selects contractility and stiffness as important parameters for describing strain in AC mutation carriers. The parameter SfAct scales the active force generation and is a measure for contractility. The parameter k1 scales the passive stress nonlinearly and is a measure for stiffness. Because disease progression in AC mutation carriers has been found to be associated with functional and structural myocardial changes (e.g. fibro-fatty replacement of myocytes [1], altered calcium handling [24] and fibrosis [25]), contractility and stiffness are likely to change during disease progression. Activation delay *dT* is in this model defined as the difference in time of activation between the segments and the IVS, whereas the activation of IVS is set to be the model's atrioventricular delay. Abnormal electrical activation is found in AC subjects [26], and, therefore, it is important to include it in the final subset. It is known that altered activation delay is related to AC disease. The remaining four parameters in the final subset par16 were heart rate, cardiac output, relative systole duration and atrioventricular delay. All these parameters have an influence on ventricular mechanics during both ejection and filling (preload) and are, therefore, important for patient-specific modelling of strain.

The parameter subset reduction is applied to the CircAdapt model, which has a low computational cost. The final parameter subset is inherent to the CircAdapt model, although it could give insight in which tissue properties should indicate to model AC disease substrates in more complex models such as three-dimensional electro-mechanical cardiac models [27,28]. These models are computationally more expensive compared to the CircAdapt model, so applying our parameter reduction framework would increase the computational cost and patient-specific parameter would be challenging. These more complex models, however, could give more insight in the mechanisms playing a role on a more local scale.

### (e) Limitations

In this study, we compare measured longitudinal strain with simulated strain along the myofibre. Because RV subendocardial myofibres are predominantly directed longitudinally [29], it is assumed that any potential difference between the two is negligible.

The objective function to minimize the error between measured and modelled strain is an important choice for fit quality and computational time. In this study, we did not investigate how to optimize the objective function, and, therefore, we used the area between measured and modelled strain curves. By using this objective function, we did not correct for any measurement errors, such as time-misregistration between the four echo views, beat-to-beat variability or inter- and intra-observer variability. Future studies could investigate how to design an objective function using strain indices or other measurements, such as valve timings, blood flow velocity or ejection fraction, to develop a more efficient fitting algorithm with an objective function invariant of measurement uncertainties. This should result in potentially better fits.

### (f) Future work

This study focuses on the development of a platform enable to reduce the number of model parameters while conserving the model's ability to simulate measured strain and is not designed to draw any conclusions on the clinical applications of fitting of deformation curves. Future work will focus on optimizing the parameter estimation algorithm regarding computational time and assess the accuracy and precision of the fits. This allows us to apply parameter estimation on a larger cohort which might expand our knowledge about the disease and its progression.

Accuracy of the parameter estimation algorithm could be estimated by repeating the algorithm to determine the precision and by using synthetic data for determining trueness. Both are assessable using the parameter estimation framework. Using ground truth measurements for determining trueness instead of synthetic data is more difficult or even impossible. Firstly, because we used a lumped parameter model, whereby the parameters are the lumped effect of the tissue, and, secondly, because these ground truth measurements are challenging to obtain. While estimated regional mechanical activation delays can potentially be compared to non-invasive or invasive electrical mapping data, it is challenging to compare regional passive and contractile tissue function parameters to ground truth measurements. Because these tissue properties are not directly measurable, our inverse modelling approach could give more insight into the underlying substrate.

Other measurements, such as voluminal information or valvular flows, may have added value to our framework and might improve accuracy and precision. However, this requires a reassessment of parameter sensitivity and identifiability, and more parameters might remain in the final parameter subset increasing the complexity of parameter estimation. Future work will show whether sufficient predicting information is captured from strain, or whether more complexity is necessary.

Although recent studies have demonstrated good reproducibility of echocardiographic RV strain measurements [5,30,31], future studies should quantify the uncertainty of the parameter estimation caused by inter- and intra-observer variability of RV strain measurements.

In this cohort, neglecting LVfw and IVS parameters (from par23 to par16) only had a small influence on parameter estimation. However, by not estimating LVfw and IVS tissue properties, no abnormalities in these walls could be found. Estimating these tissue properties might be of interest in future studies because there can be LV involvement in later stadia of the disease and in other cohorts with other mutations [32]. Including LV involvement might disagree with the assumption of homogeneous mechanics in the LVfw and IVS wall should also be reassessed. This framework should be used for re-evaluating the parameter subset.

## 5. Conclusion

To identify the set of parameters needed for patient-specific modelling of RV myocardial disease in AC mutation carriers using the CircAdapt model, we set up a framework for parameter subset reduction. Using MSM and qMC, we successfully reduced the number of parameters from 110 to 16. The final subset includes regional tissue contractility, passive stiffness, activation delay and wall size. By estimating these parameters using PSO, the CircAdapt model was still able to accurately simulate strain in AC-related mutation carriers. Future work should use strain indices instead of the whole curve to optimize the fitting algorithm. This will allow us to apply the algorithm on larger cohorts, as well as to relate estimated parameters to disease progression and outcome.

## Supplementary Material

Supplemental Material

## Supplementary Material

Figures Supplemental Material

## Supplementary Material

Source Code

## Data accessibility

The source code supporting this article have been uploaded as part of the electronic supplementary material.
