# Supplementary material for: Parameter subset reduction for patient-specific modelling of arrhythmogenic cardiomyopathy-related mutation carriers in the CircAdapt model
Source: Philos Trans A Math Phys Eng Sci. 2020 May 25;378(2173):20190347. doi: 10.1098/rsta.2019.0347 (PMC7287326; doi:10.1098/rsta.2019.0347)
Supplement: Supplemental Material [file rsta20190347supp1.pdf]

Supplemental Material

1. The CircAdapt sarcomere contraction model

This section is obtained from Walmsley et al.: The MultiPatch Model, supplemental material to Walmsley et al. (2015): Fast Simulation of Mechanical Heterogeneity in the Electrically Asynchronous Heart Using the MultiPatch Module (1).

The tissue parameters in the final subset, par16, are:

- *Contractility SfAct ($\sigma_{f,act}$, Equation 13)*
- *Stiffness k1 (k_1, Equation 16)*
- *Activation Delay dT (t_{act} in Equation 10)*
- *Reference Segment Area AmRef (A_{ref} in Equation 25)*

The contraction model currently used in CircAdapt is a modified Hill model based upon the one presented by Lumens *et al[2]*. The model aims to reproduce basic properties of length dependent activation in cardiac tissue [3,4]. The fibre stress is determined by the rise of contractility in the fibre (representing density of cross bridge formation) and the fibre strain. The fibre model is divided into an active and passive stress component, with the active stress arising from myofibre contraction, and the passive stress component arising from the soft tissue deformation of the myocardium.

The current myofibre strain is used to compute the sarcomere length in the model. In CircAdapt, natural myofibre strain ε_f in a patch is defined as

$$\varepsilon_f = \ln \frac{L_s}{L_{s,\mathrm{Ref}}}, \quad (1)$$

where L_s is the total sarcomere length, and $L_{s,Ref}$ is the reference sarcomere length of 2µm. From the strain we can therefore calculate the sarcomere length as

$$L_s = L_{s,\mathrm{ref}} \exp(\varepsilon_f) \quad (2)$$

1.1. *Fibre active stress*

The fibre active stress is determined by a modified Hill model controlled by two variables, the intrinsic sarcomere length L_{si} and the contractility *C*. The governing equation for L_{si} is

$$\frac{dL_{si}}{dt} = v_{\max} \left(\frac{L_s - L_{si}}{L_{se,\mathrm{iso}}} - 1 \right), \quad (3)$$

where $L_s - L_{si}$ is the length of the series elastic element in the Hill model, and $L_{se,iso}$ is the length of the series elastic element during isovolumetric contraction. The length of the series elastic element represents the deformation of the sarcomere due to stretch of cross bridges under mechanical load during contraction.

Contractility is a phenomenological parameter representing the density of cross bridge formation within the fibres in the current patch. The contractility is determined by the following differential equation,

$$\frac{dC}{dt} = \frac{1}{\tau_{\mathrm{rise}}} C_L(L_{si})\, F_{\mathrm{rise}}(t) - \frac{1}{\tau_{\mathrm{decay}}} C\, \mathrm{g(X)}, \quad (4)$$

where,

$$\tau_{\mathrm{rise}} = 0.55\, T_r t_A, \quad (5)$$

$$\tau_{\mathrm{decay}} = 0.33\, T_d t_A. \quad (6)$$

T_r and T_d are constants, and t_A is the duration of activation of the fibre. t_A depends on the sarcomere extension,

$$t_A = 0.65 + 1.057\,\frac{L_{si}}{L_{si,0}}. \tag{7}$$

C_L describes the increase in cross bridge formation with intrinsic sarcomere length due to an increase in available binding sites,

$$C_L(L_{si}) = \tanh\left(4\left(L_{si} - L_{si,0}\right)^2\right). \tag{8}$$

$F_{rise}(t)$ is a phenomenological representation of the rate of cross bridge formation within the patch,

$$F_{\mathrm{rise}}(t) = 0.02\,x^3(8-x)^2\exp(-x), \tag{9}$$

where

$$x(t) = \min\left(8, \max\left(0, \frac{t_{\mathrm{c}}}{\tau_{\mathrm{rise}}}\right)\right), \tag{10}$$

and $t_c = t - t_{act}$, where t_{act} is the time of onset of activation of the patch, *i.e.* the time at which the first myocytes within the patch begin to form cross bridges in response to electrical activation.

The decay term in equation (4) gives an exponential decay in the contractility. This decay is delayed by the term *g(X)*. The term *g(X)* is an approximation of the function *tanh(X)* using a sine curve to ensure that it takes value 0 or 1 outside of the region where it exhibits a large change,

$$\mathrm{g(X)} = 0.5 + 0.5\,\sin\left(\mathrm{sign}(X)\min\left(\frac{\pi}{2}, \mathrm{abs}(X)\right)\right), \tag{11}$$

where,

$$X = \frac{t_c - t_A}{\tau_d}. \tag{12}$$

The effect of the formulation for contractility is as follows: as the chamber wall is stretched by an expanding volume of blood, the series elastic element (L_{se}) lengthens, causing a corresponding lengthening of the contractile element (L_{si}). Given an onset of cross bridge formation in response to electrical excitation of parts of the patch at time t_{act}, the contractility *C* begins to rise according to $F_{rise}(t)$. The longer the contractile element L_{si}, the greater both the duration of the contractile phase (equation (7)) and the rate of increase in contractility (equation (8)) are. Once the duration of activation, t_{act}, is over, the contractility begins to decay exponentially.

We use the following equations to convert contractility and sarcomere length into actively generated fibre stress $\sigma_{f,actT}$ within a patch,

$$\sigma_{f,\mathrm{actT}} = \sigma_{f,\mathrm{act}} C(L_{si} - L_{si,\mathrm{ref}})\frac{L_{se}}{L_{se,\mathrm{iso}}}, \tag{13}$$

where $\sigma_{f,act}$ is a parameter and L_{se} / $L_{se,iso}$ is the extension of the series elastic element. Hence the actively generated fibre stress is determined by the stretching of the myosin heads in response to sarcomere shortening multiplied by the number of cross bridges formed, which is the contractility multiplied by the sarcomere extension from reference ($C\,(L_{si} - L_{si,ref})$).

1.2. Fibre passive stress

Passive deformation of the soft tissue making up the myocardium will also generate stress within the walls, $\sigma_{f,pasT}$. In CircAdapt, this is considered to be a passive stress in the fibres in each patch. This contains two components, the stress arising from the myocytes themselves

due to internal structures such as titin anchoring to the Z disc ($\sigma_{f,tit}$), and the stress arising from the extracellular matrix surrounding the myocytes ($\sigma_{f,ECM}$). Hence,

$$\sigma_{f,pasT} = \sigma_{f,tit} + \sigma_{f,ECM}. \tag{14}$$

The extension of the cells for the passive stress calculation $\lambda_{s,pas}$ is done relative to a different reference length $L_{s0,pas}$ as follows,

$$L_{s,pas} = \frac{L_{s0}}{L_{s0,pas}} \exp(e_f). \tag{15}$$

The ECM is modelled as being stiffer than the contribution due to cellular structures such as titin,

$$\sigma_{f,ECM} = 0.0349\, \sigma_{f,pas} \left({\lambda_{s,pas}}^{k_1} - 1\right), \tag{16}$$

where $\sigma_{f,pas}$ is a parameter.

The passive stress in the patch due to cellular structures such as titin is modelled as being softer than the ECM, and is governed by the following equation

$$\sigma_{f,tit} = 0.01\ \sigma_{f,act} \left({\lambda_{s,pas}}^{k_2} - 1\right) \tag{17}$$

Where the parameter k is given by

$$k_2 = \frac{2L_{s,ref}}{dL_{s0,pas}}, \tag{18}$$

and $dL_{s0,pas}$ is a parameter. Using equations (13) and (14) we then arrive at the following expression for fibre stress within a patch,

$$\sigma_f = \sigma_{f,actT} + \sigma_{f,pasT}. \tag{19}$$

1.3. *Derivative of fibre stress with respect to fibre strain*

As described in the main article, calculating the compliance in a patch requires the stiffness $\frac{d\sigma_f}{de_f}$. We see from equations (15), (16) and (17) that,

$$\frac{d\sigma_{f,pasT}}{de_f} = 0.349\, \sigma_{f,pas}\, {\lambda_{s,pas}}^{k_1} + 0.01\, k\, \sigma_{f,act} {\lambda_{s,pas}}^{k_2}\ . \tag{20}$$

and we can calculate $\frac{d\sigma_{f,actT}}{de_f}$ using equation (13), equation (2), and the relation $L_{se} = L_s - L_{si}$,

$$\frac{d\sigma_{f,actT}}{de_f} = \sigma_{f,\text{act}} C(L_{si} - L_{si,\text{ref}}) \frac{L_s}{L_{se,\text{iso}}}. \tag{21}$$

We then have,

$$\frac{d\sigma_f}{de_f} = \frac{d\sigma_{f,actT}}{de_f} + \frac{d\sigma_{f,pasT}}{de_f}\ . \tag{22}$$

1.4. *Conservation of energy*

CircAdapt connects wall tension T and wall area A to fibre stress σ_f and strain ε_f through the law of conservation of energy. Due to the transmural averaging assumptions in CircAdapt, changes in wall tension and area within a patch or wall must correspond to changes in fibre stress and strain throughout the volume of that patch or wall,

$$T\, dA = V_w\, \sigma_f\, d\varepsilon_f \tag{23}$$

Hence,

$$T = V_w\, \sigma_f\, \frac{d\varepsilon_f}{dA} \tag{234}$$

From the relation between fibre stress and wall area (Eq. 1 in main article), we have

$$\varepsilon_f = \frac{1}{2} \ln\left(\frac{A}{A_{Ref}}\right) \tag{245}$$

And so,

$$T = \frac{V_W\, \sigma_f}{2A} \tag{256}$$

2. Morris Screening

2.1. Additional Methods

Simulation Protocol

The input space $\Omega = \mathbb{R}^D$ was spanned by all model parameters (initially, $D = 110$) whose domain was linearly normalized to a range from 0 to 1. Each trajectory starts at a random point $\boldsymbol{a}$ on the z-level grid (z=8) and ends at point $\boldsymbol{b}$, such that $|a_i - b_i| = \frac{1}{2}\frac{z}{z-1}$.

Firstly, the simulation with the closest Euclidean distance to the reference was performed. Secondly, the closest simulation of the closest trajectory to the already finished simulations was performed. This step was repeated until each trajectory had one finished simulation. Failed simulations ran again because closer starting points could be available.

Thirdly, all simulations in each trajectory were performed. Each simulation uses the closest simulation of that trajectory as a starting point and performs one step in between. If the simulation fails again, it was done in 10 steps and if that fails, it was done in 100 steps. If this fails, the trajectory was removed. This step was repeated until all trajectories were done.

Finally, the number of trajectories was evaluated. If the number of successful trajectories was less than 1000, 500 trajectories were added. For these trajectories, the second, third, and fourth step were repeated.

2.2. Results

For each parameter i, the absolute average μ_{ij}^* of the elementary effect on output j in the first screening is shown in Figure 1. White indicates an average near zero and red indicates importance near but below one. Green indicates that the parameter has an influence on the output. To the left of the black line, no parameter had an effect on any output. Therefore, they are nonidentifiable given the defined outputs and were omitted from the parameter subset. All parameters of the pericardium and valves were omitted. Furthermore, most parameters of the circulation and atria were omitted.

Using the important parameters of the first screening, a second screening was done. The absolute average μ_{ij}^* of the elementary effect in the second screening is shown in Figure 2. Similar to the first screening, mostly parameters of the circulation and atria were omitted.

Using the important parameters of the second screening, a third screening was done. The absolute average μ_{ij}^* of the elementary effect in the second screening is shown in Figure 3. Only one parameter was irrelevant and therefore omitted. No further screening was done because the computational cost outweighed the expected parameter reduction.

Figure 4 shows the convergence check on the first screening. All three screenings did converge within the 1000 trajectories.

3. Subset Reduction

To reduce the parameter subset from par53 to par26, two steps are performed in between, i.e. par40 and par31. The fit error of all parameter subsets is shown in Figure 5. The example fits of all subjects with the subsets par53, par23, and par16 are shown in Figure 6-20.

4. References

1. Walmsley J, Arts T, Derval N, Bordachar P, Cochet H, Ploux S, et al. Fast Simulation of Mechanical Heterogeneity in the Electrically Asynchronous Heart Using the MultiPatch Module. PLoS Comput Biol. 2015;11(7):1–23.
2. Lumens J, Delhaas T, Kirn B, Arts T (2009) Three-wall segment (TriSeg) model describing mechanics and hemodynamics of ventricular interaction. Ann Biomed Eng 37: 2234-2255.
3. de Tombe PP, ter Keurs HE (1990) Force and velocity of sarcomere shortening in trabeculae from rat heart. Effects of temperature. Circ Res 66: 1239–1254.
4. ter Keurs HE, Rijnsburger WH, van Heuningen R, Nagelsmit MJ (1980) Tension development and sarcomere length in rat cardiac trabeculae. Evidence of length-dependent activation. Circ Res 46: 703-714.
