# Supplementary material for: Parameter subset reduction for patient-specific modelling of arrhythmogenic cardiomyopathy-related mutation carriers in the CircAdapt model
Source: Philos Trans A Math Phys Eng Sci. 2020 May 25;378(2173):20190347. doi: 10.1098/rsta.2019.0347 (PMC7287326; doi:10.1098/rsta.2019.0347)
Supplement: Figures Supplemental Material [file rsta20190347supp2.pdf]

parname	lower	upper	par53	par40	par31	par26	par23	par16
Ls0Pas LA	1.7μm	1.9μm	✓					
SfAct LV	96kPa	144kPa	✓	✓	✓	✓	✓	
k1 LV	8	12	✓	✓	✓	✓	✓	
dT LV	-0.025ms	0.025ms	✓	✓	✓	✓	✓	
vMax LV	5.6$\mu m/ms$	8.4$\mu m/ms$	✓					
TR LV	0.2ms	0.3ms	✓	✓	✓			
TD LV	0.2ms	0.3ms	✓					
VWall LV	76.808mL	115.212mL	✓	✓				
AmRef LV	7840mm^2	11760mm^2	✓	✓	✓	✓	✓	
SfAct SV	96kPa	144kPa	✓	✓	✓	✓	✓	
k1 SV	8	12	✓	✓	✓	✓	✓	
Ls0Pas SV	1.7μm	1.9μm	✓	✓	✓			
TD SV	0.2ms	0.3ms	✓					
VWall SV	25.888mL	38.832mL	✓	✓				
AmRef SV	3920mm^2	5880mm^2	✓	✓	✓	✓	✓	
SfAct RVapex	96kPa	144kPa	✓	✓	✓	✓	✓	✓
SfPas RVapex	18kPa	27kPa	✓	✓	✓	✓		
k1 RVapex	8	12	✓	✓	✓	✓	✓	✓
dT RVapex	-0.025ms	0.025ms	✓	✓	✓	✓	✓	✓
Ls0Pas RVapex	1.7μm	1.9μm	✓	✓	✓			
vMax RVapex	5.6$\mu m/ms$	8.4$\mu m/ms$	✓	✓				
TR RVapex	0.2ms	0.3ms	✓	✓				
TD RVapex	0.2ms	0.3ms	✓					
VWall RVapex	16.792mL	25.188mL	✓					
AmRef RVapex	3440mm^2	5160mm^2	✓	✓	✓	✓	✓	✓
dLsPas RVapex	0.48	0.72	✓					
SfAct RVmid	0kPa	120kPa	✓	✓	✓	✓	✓	✓
SfPas RVmid	11kPa	228kPa	✓	✓	✓	✓		
k1 RVmid	5	50	✓	✓	✓	✓	✓	✓
dT RVmid	-0.025ms	0.075ms	✓	✓	✓	✓	✓	✓
Ls0Pas RVmid	1.7μm	1.9μm	✓	✓	✓			
vMax RVmid	0.01$\mu m/ms$	7$\mu m/ms$	✓	✓				
TR RVmid	0.2ms	0.3ms	✓	✓				
TD RVmid	0.2ms	0.3ms	✓					
VWall RVmid	16.792mL	41.98mL	✓					
AmRef RVmid	3440mm^2	5160mm^2	✓	✓	✓	✓	✓	✓
dLsPas RVmid	0.01	1.2	✓					
SfAct RVbase	0kPa	120kPa	✓	✓	✓	✓	✓	✓
SfPas RVbase	11kPa	228kPa	✓	✓	✓	✓		
k1 RVbase	5	50	✓	✓	✓	✓	✓	✓
dT RVbase	-0.025ms	0.075ms	✓	✓	✓	✓	✓	✓
Ls0Pas RVbase	1.7μm	1.9μm	✓	✓	✓			
vMax RVbase	0.01$\mu m/ms$	7$\mu m/ms$	✓	✓				
TR RVbase	0.2ms	0.3ms	✓	✓				
TD RVbase	0.2ms	0.3ms	✓					
VWall RVbase	16.792mL	25.188mL	✓					
AmRef RVbase	3440mm^2	5160mm^2	✓	✓	✓	✓	✓	✓
dLsPas RVbase	0.01	1.2	✓					
p0	9.76kPa	14.64kPa	✓	✓				
q0	4.08L/min	6.12L/min	✓	✓	✓	✓	✓	✓
tCycle	0.6s	1.25s	✓	✓	✓	✓	✓	✓
dTauAv	-0.025ms	0.025ms	✓	✓	✓	✓	✓	✓
TimeFac	0.5	1.5	✓	✓	✓	✓	✓	✓
parname	lower	upper	par53	par40	par31	par26	par23	par16

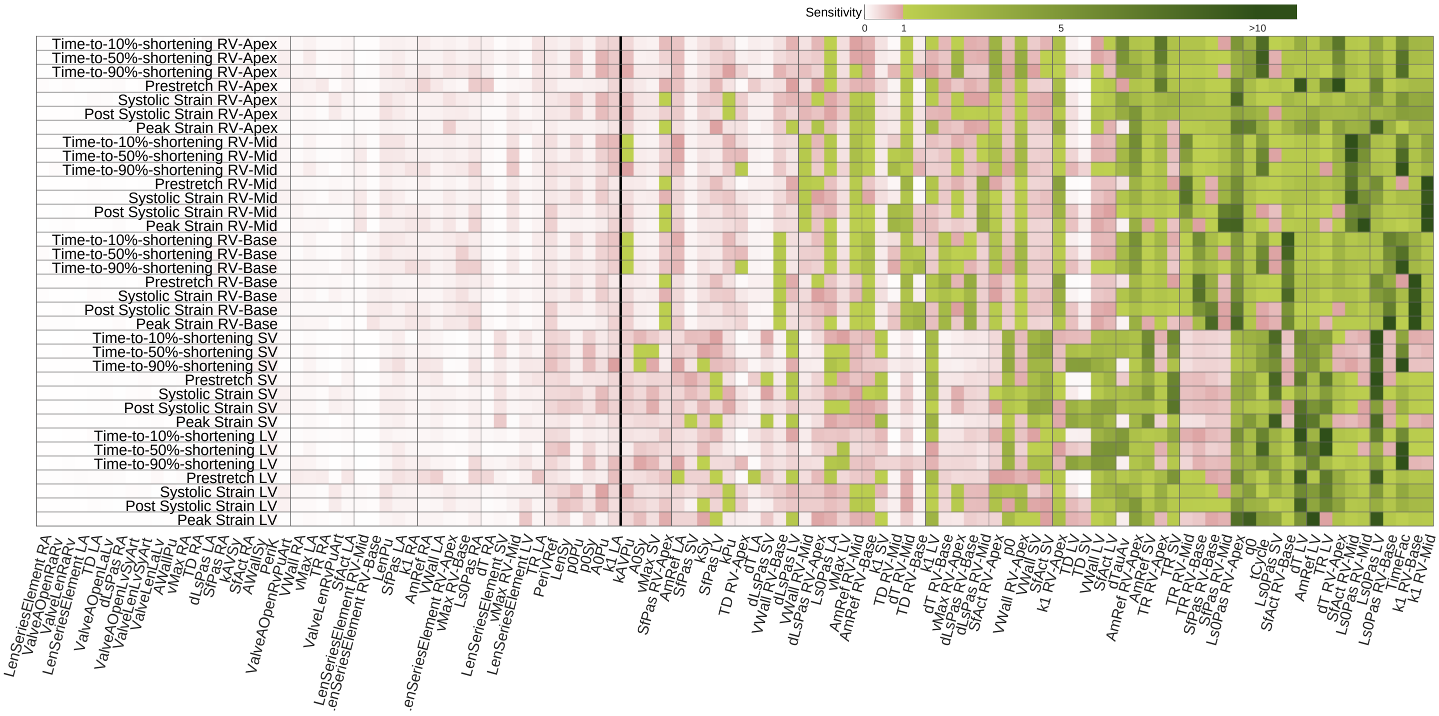

Figure 1: Parameter importance according to Morris Screening in the first screening. Vertically, all strain indices are shown. Important is normalized to the mean effect of the strain index. Non-important parameters with sensitivity below one are shown in white/red. Important parameters with sensitivity above one are shown in green. Parameters are ranked on maximum sensitivity to an output. All important parameters (at least one effect > 1) are shown on the right of the thick vertical line.

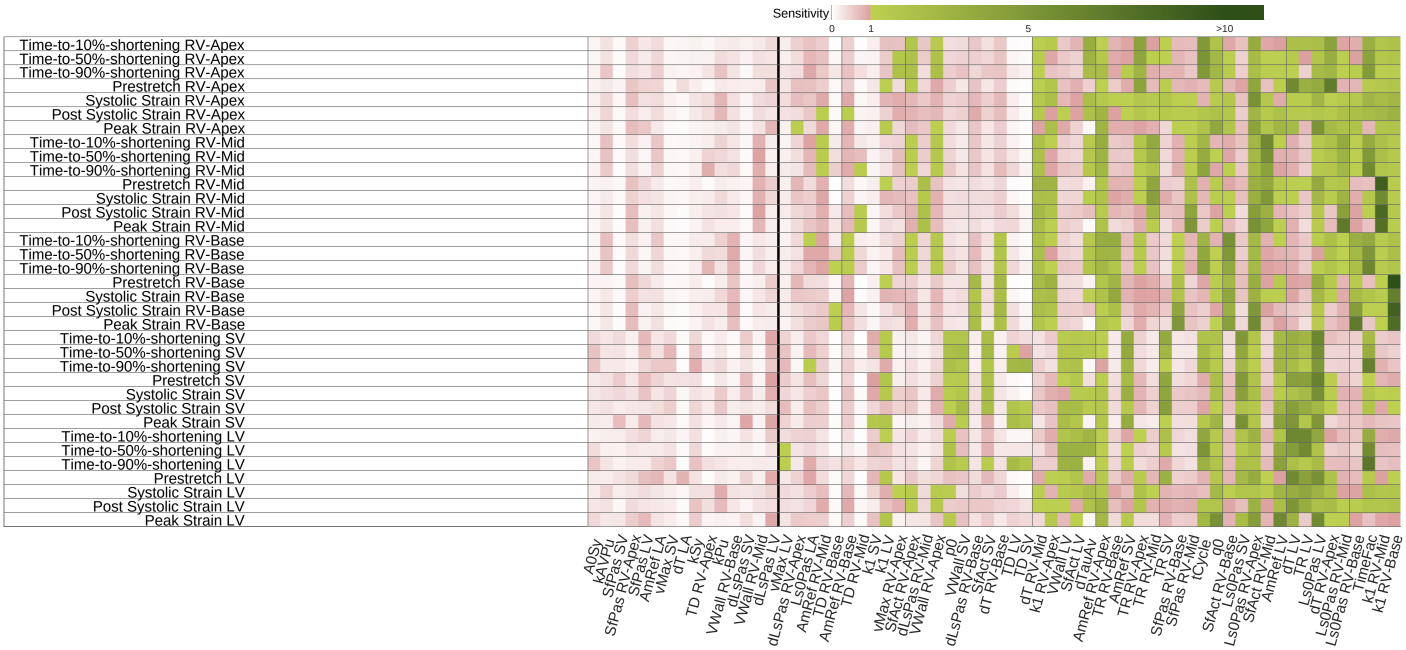

Figure 2: Parameter importance according to Morris Screening in the second screening. Vertically, all strain indices are shown. Important is normalized to the mean effect of the strain index. Non-important parameters with sensitivity below one are shown in white/red. Important parameters with sensitivity above one are shown in green. Parameters are ranked on maximum sensitivity to an output. All important parameters (at least one effect > 1) are shown on the right of the thick vertical line.

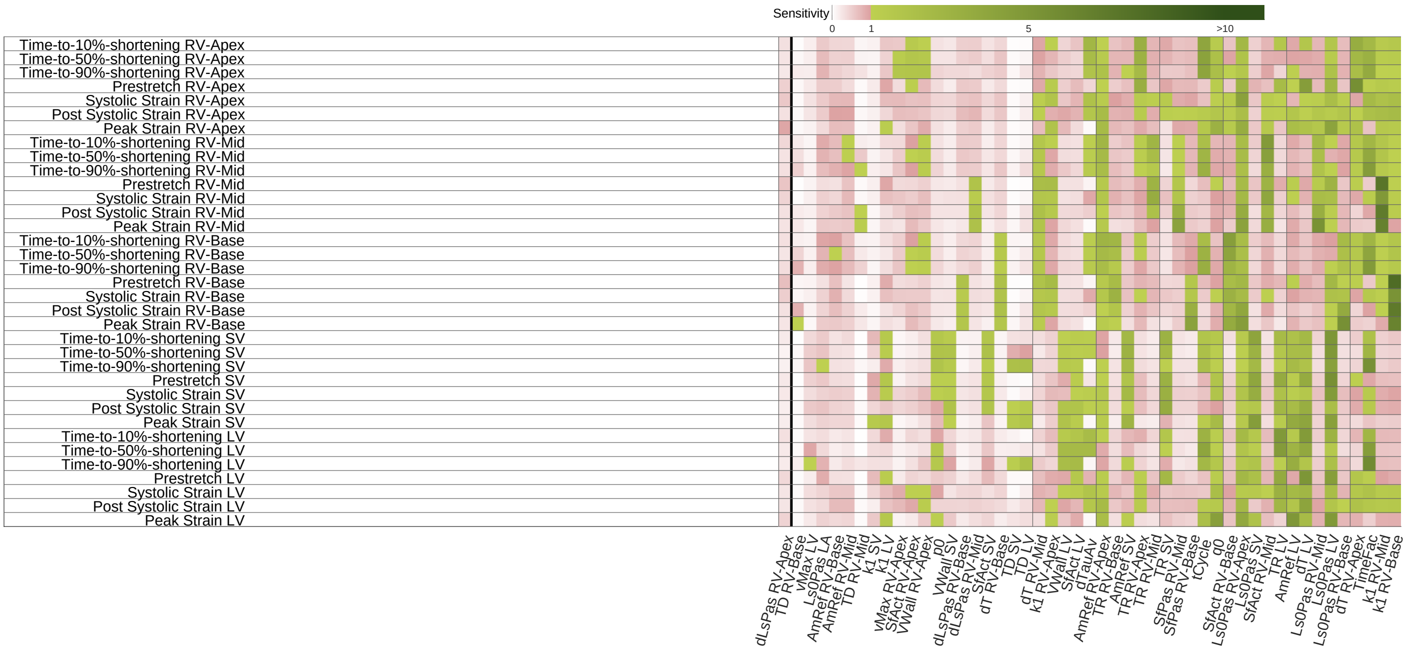

Figure 3: Parameter importance according to Morris Screening in the third screening. Vertically, all strain indices are shown. Important is normalized to the mean effect of the strain index. Non-important parameters with sensitivity below one are shown in white/red. Important parameters with sensitivity above one are shown in green. Parameters are ranked on maximum sensitivity to an output. All important parameters (at least one effect > 1) are shown on the right of the thick vertical line.

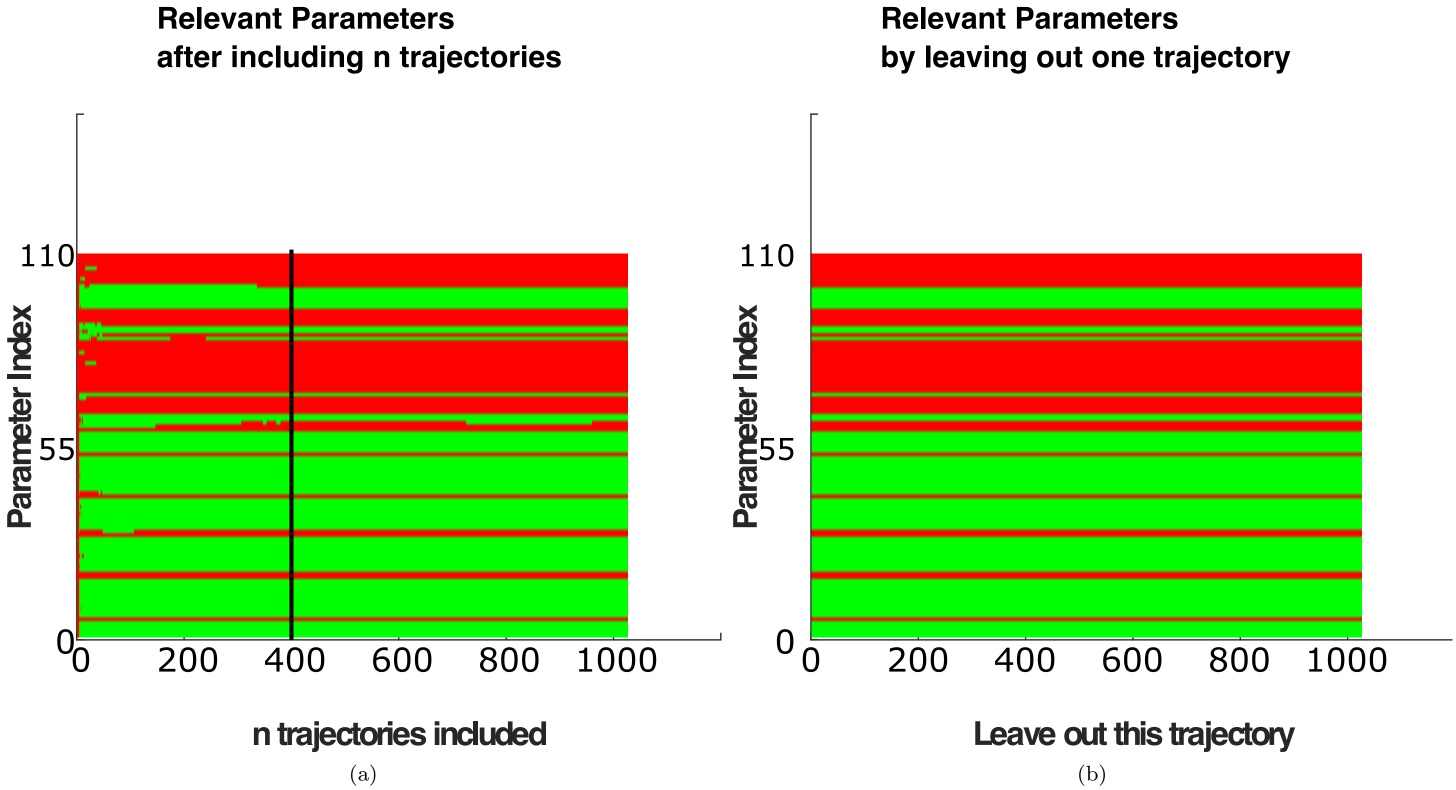

Figure 4: Convergence of Morris Screening of the first screening. (a) Convergence over time: After the first 400 random trajectories (black verticle line), only one parameter changes in importance such that omitting might be questionable. This parameter, which is k1 of the left atrium, is excluded in the next screening. For all screenings, convergence was validated. (b) Leave one out method. Each parameter is left out once, and the reduced subset is compared. For all, the reduced subset contained the same parameters, which validated the convergence.

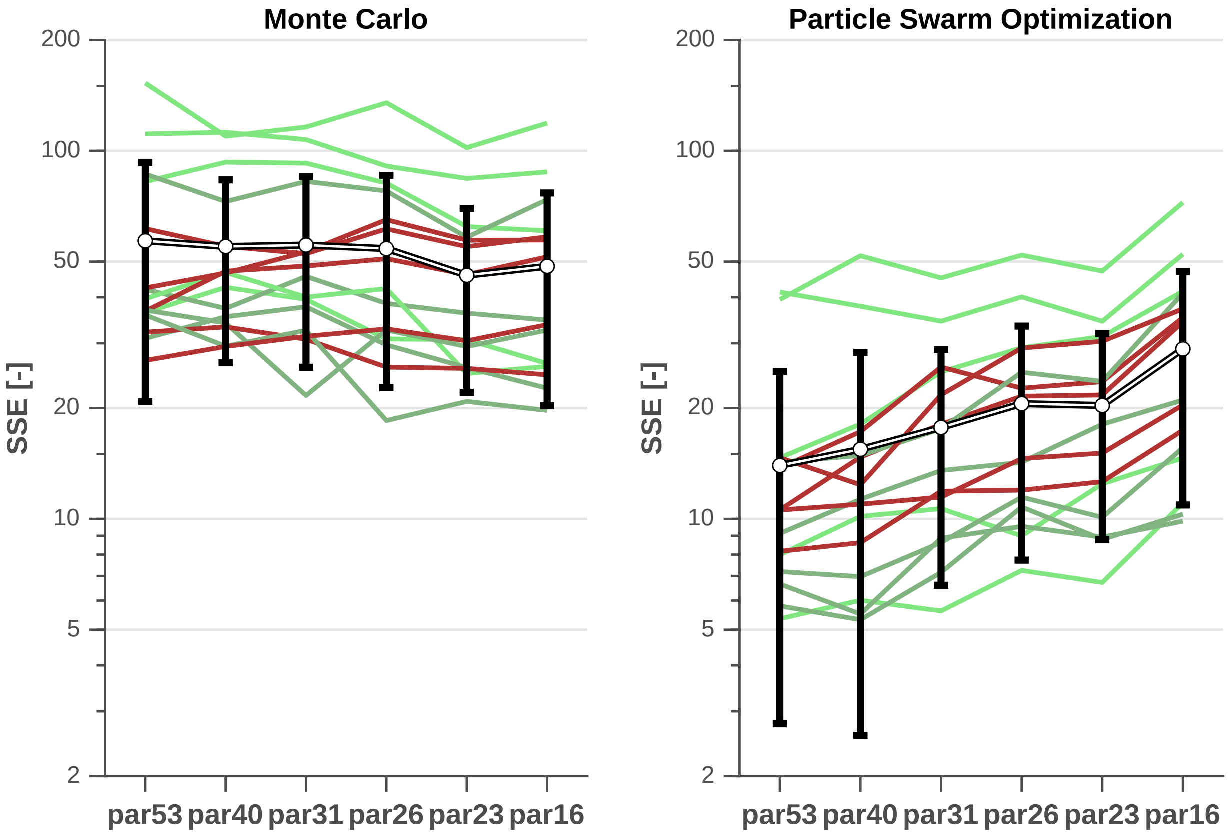

Figure 5: Minimum fit error (E_{SS}) after quasi-Monte Carlo (left) and after Particle Swarm Optimization (right) of all subsets, where parX indicates the number of parameters included. Green lines indicates subjects in the concealed stage, the light and dark red lines indicate subjects in the electrical and structural stage. Black-white lines show the average summed squared error of all subjects including standard deviation.

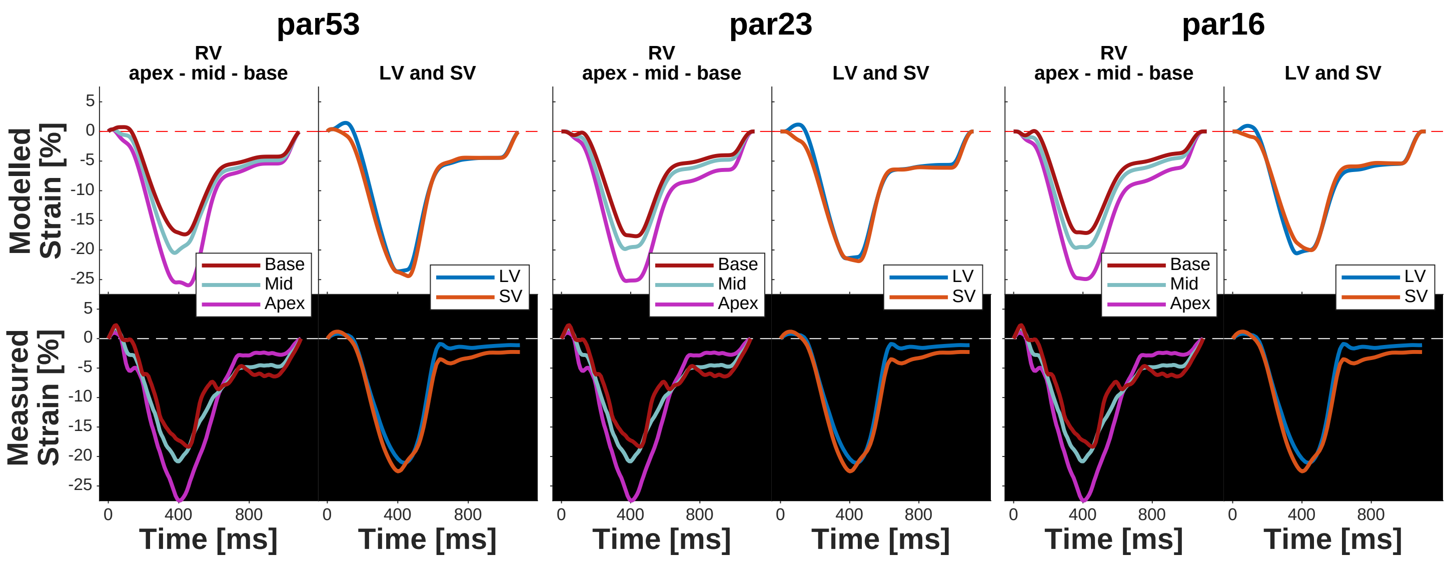

Figure 6: Fits of patient 1

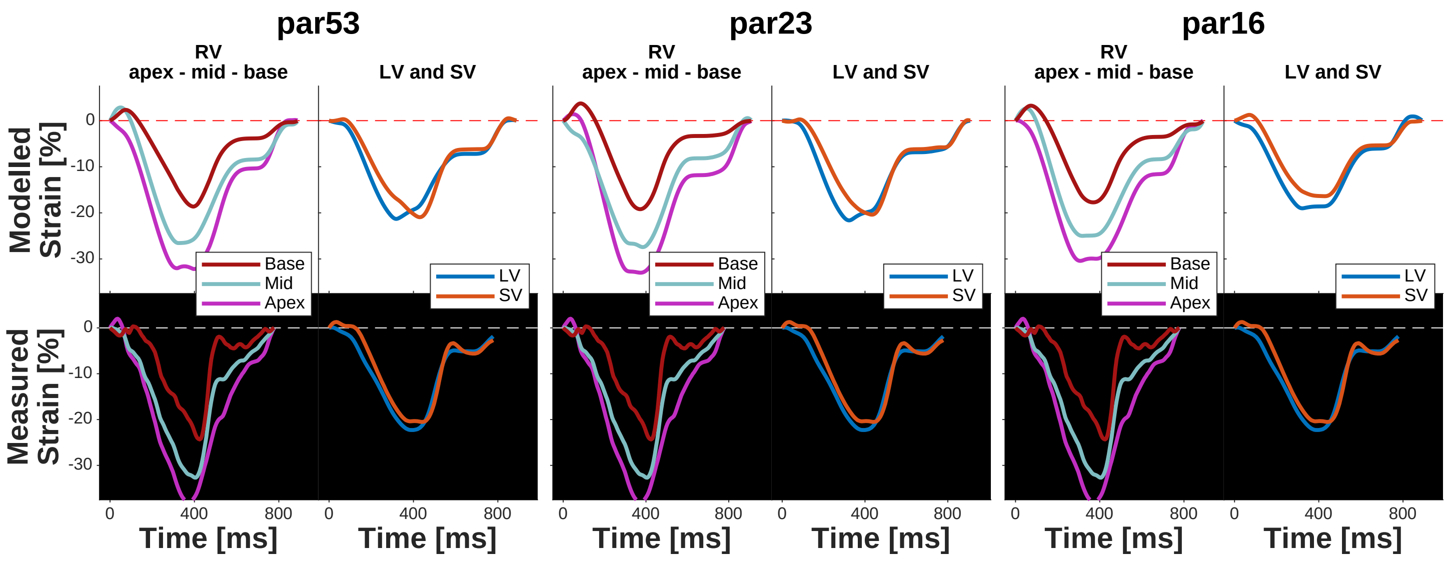

Figure 7: Fits of patient 2

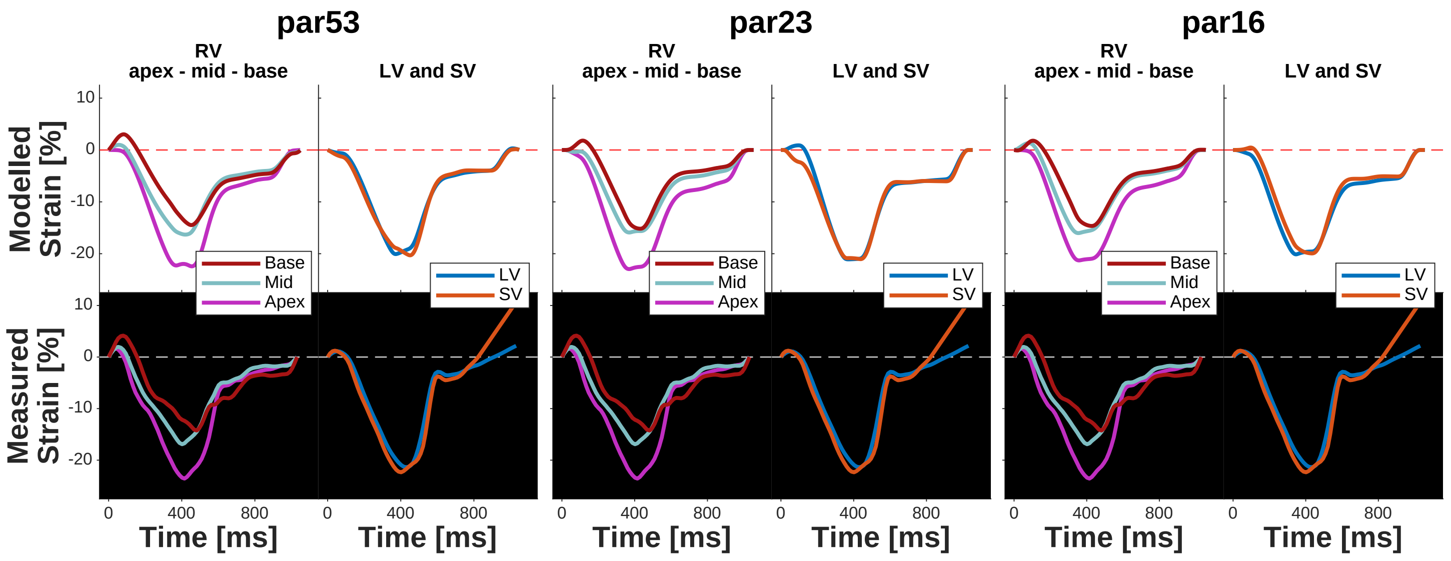

Figure 8: Fits of patient 3

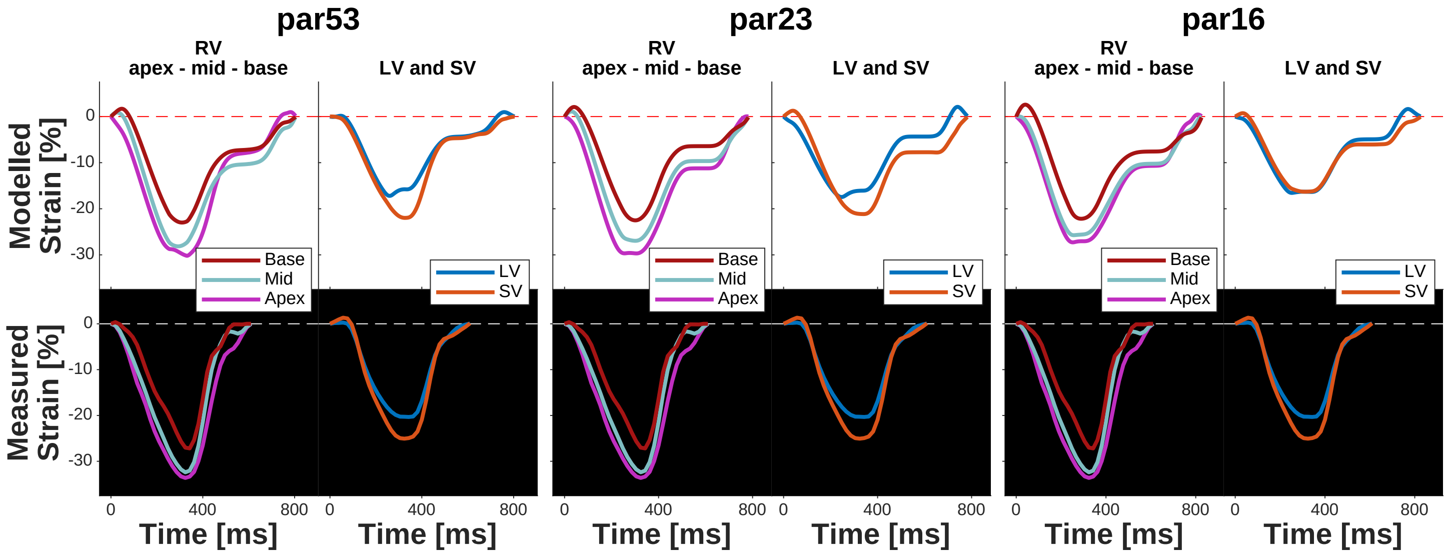

Figure 9: Fits of patient 4

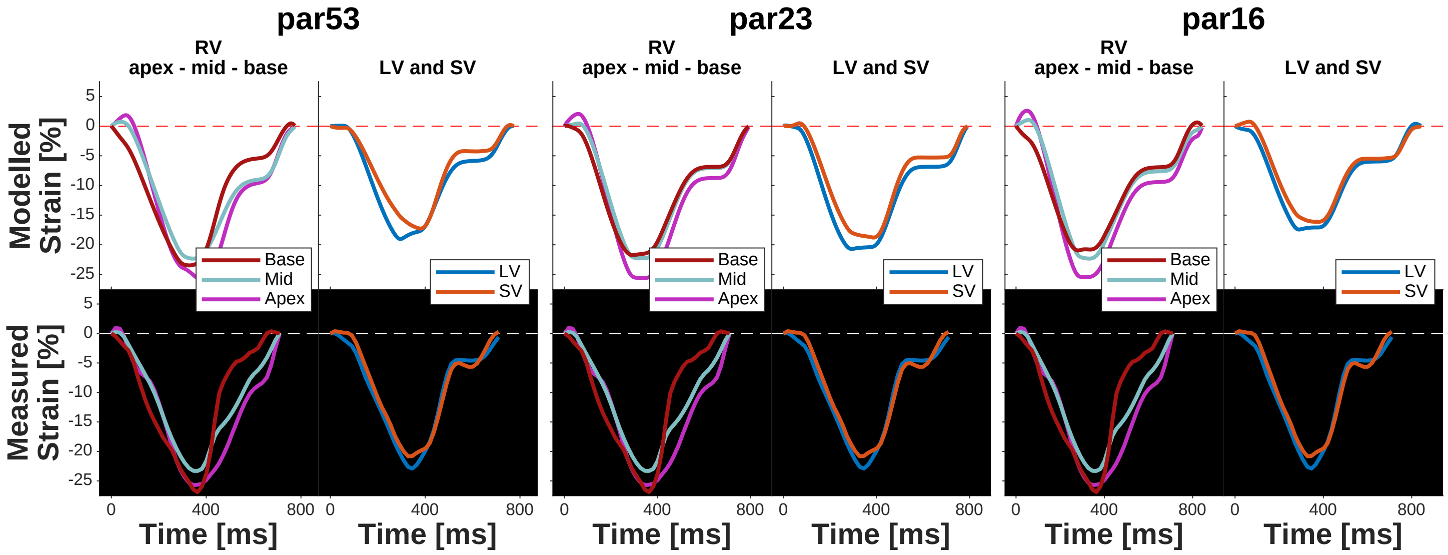

Figure 10: Fits of patient 5

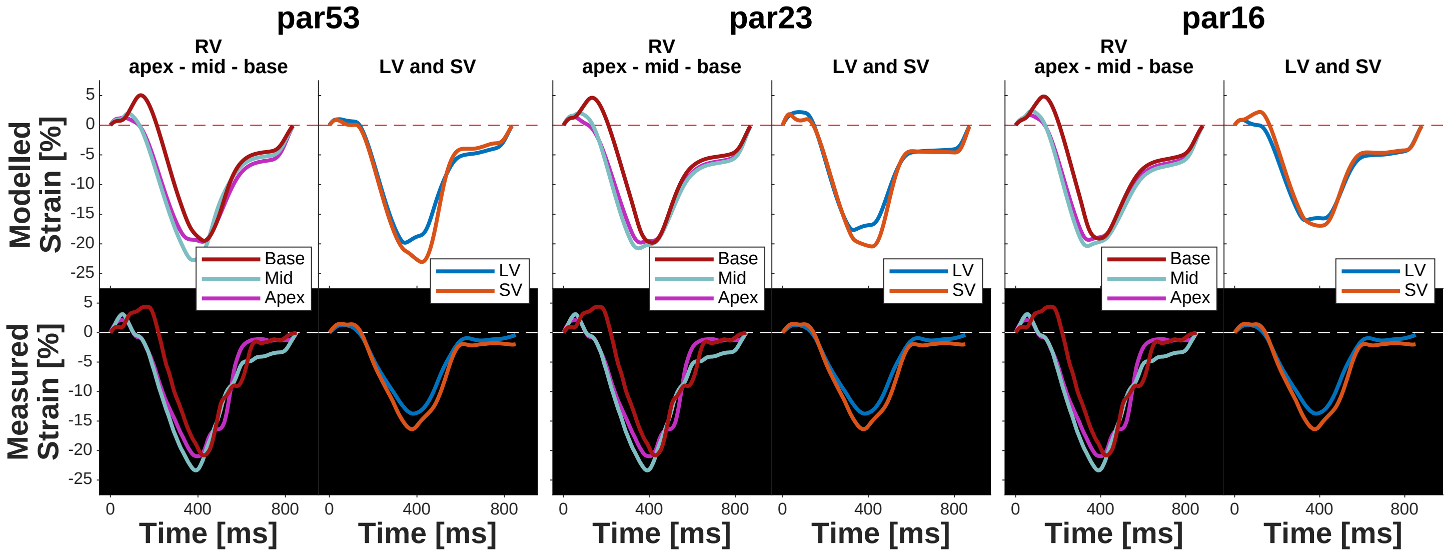

Figure 11: Fits of patient 6

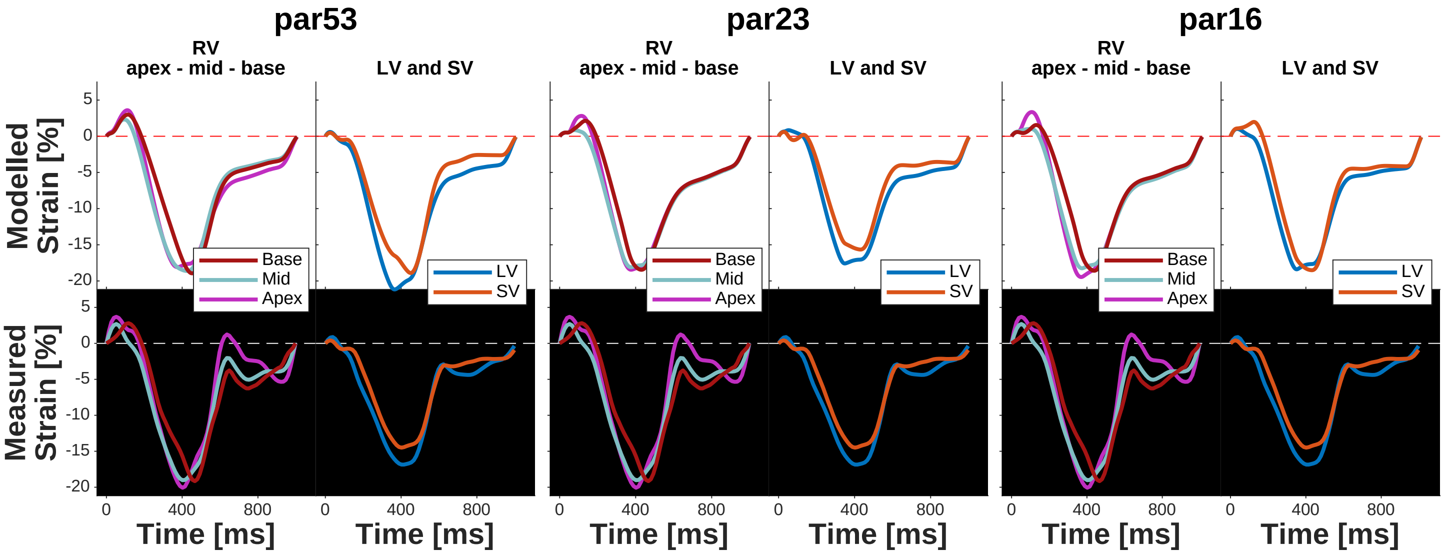

Figure 12: Fits of patient 7

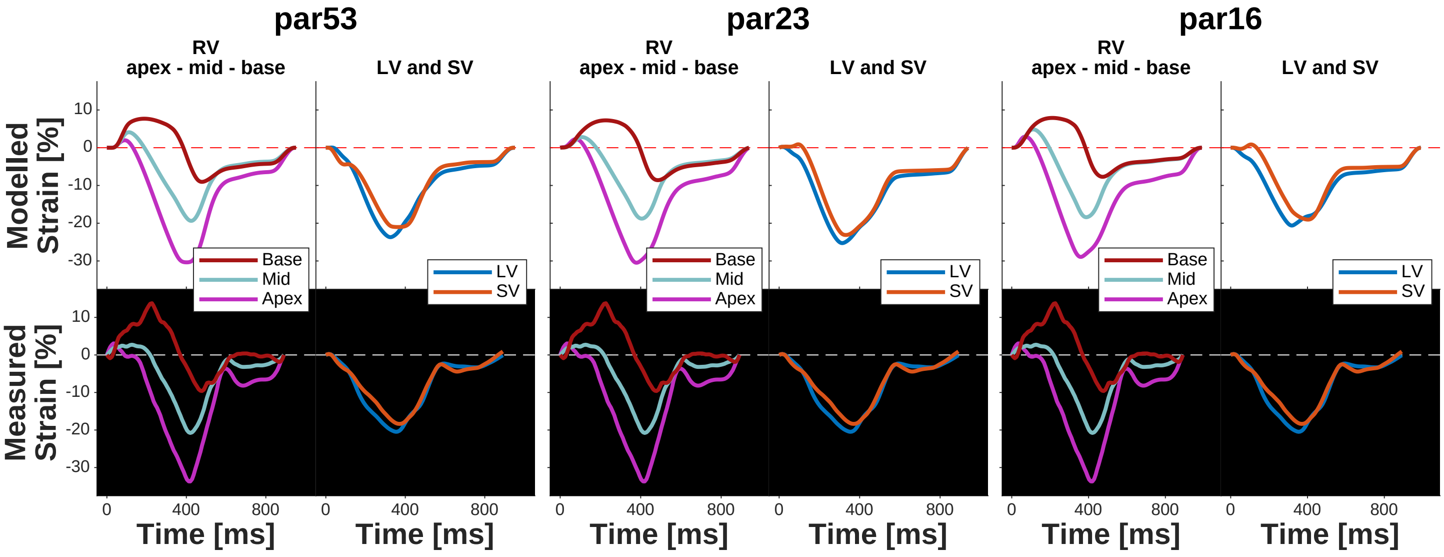

Figure 13: Fits of patient 8

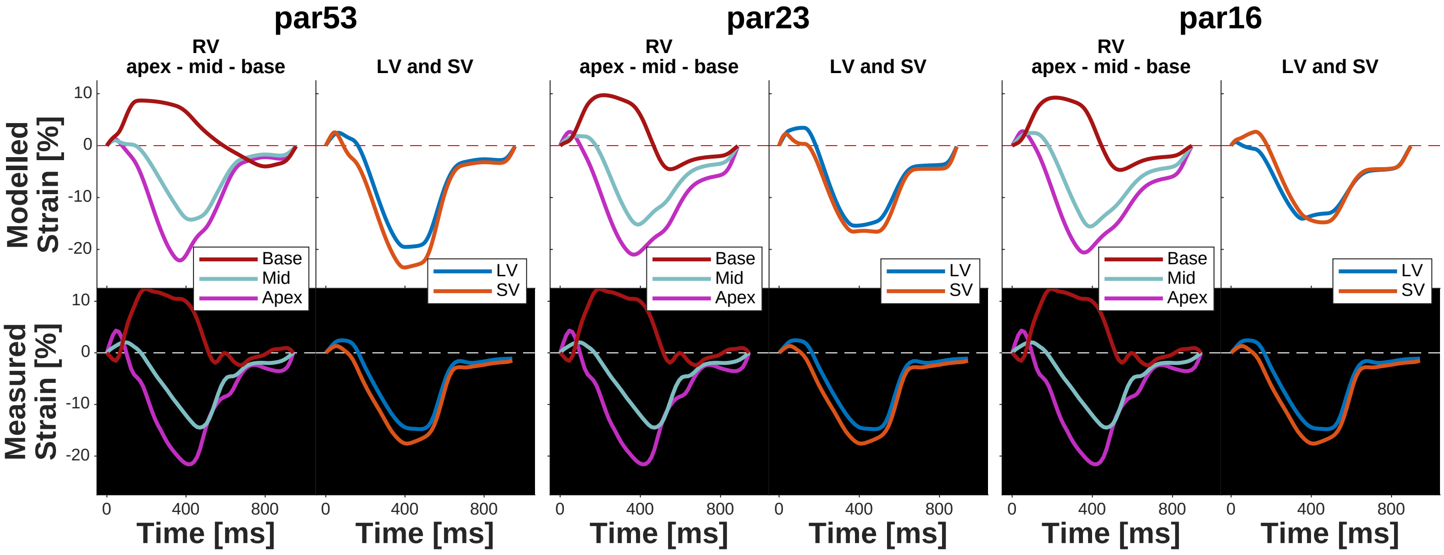

Figure 14: Fits of patient 9

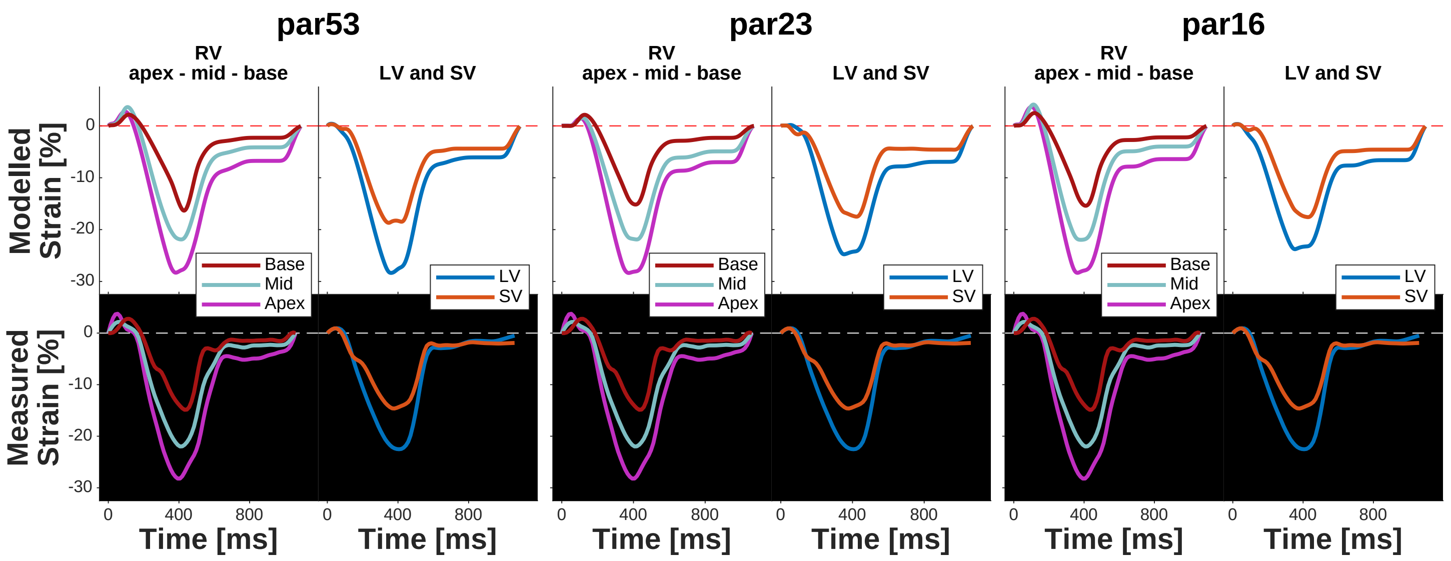

Figure 15: Fits of patient 10

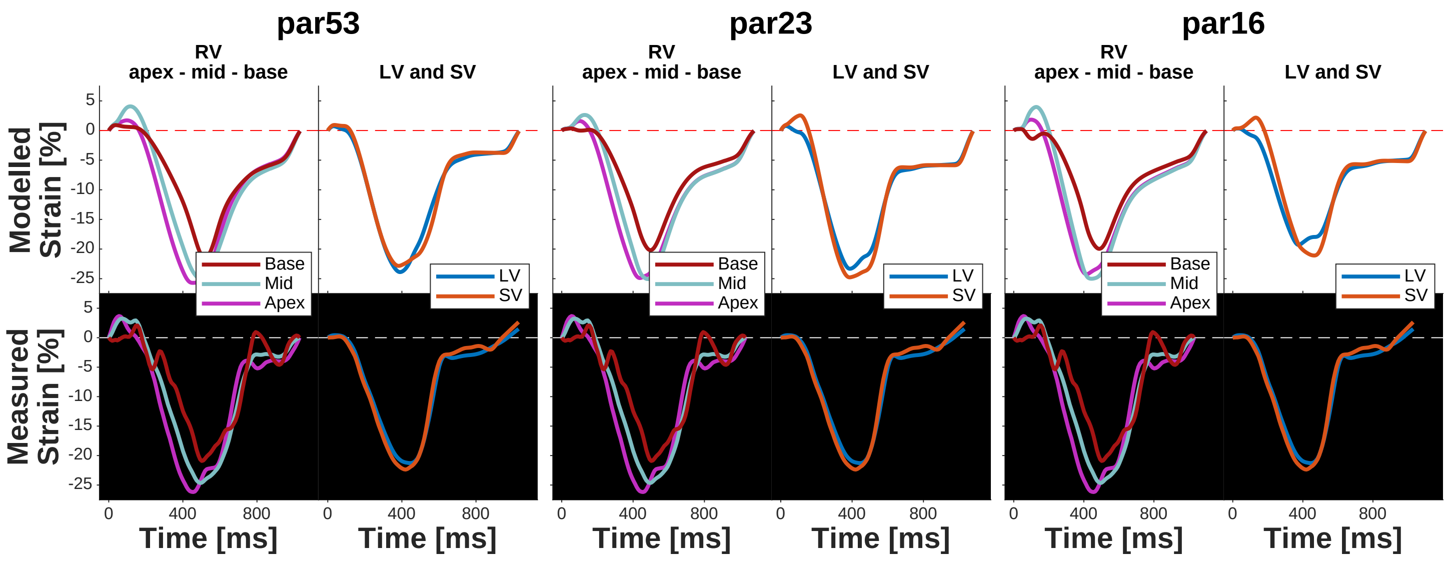

Figure 16: Fits of patient 11

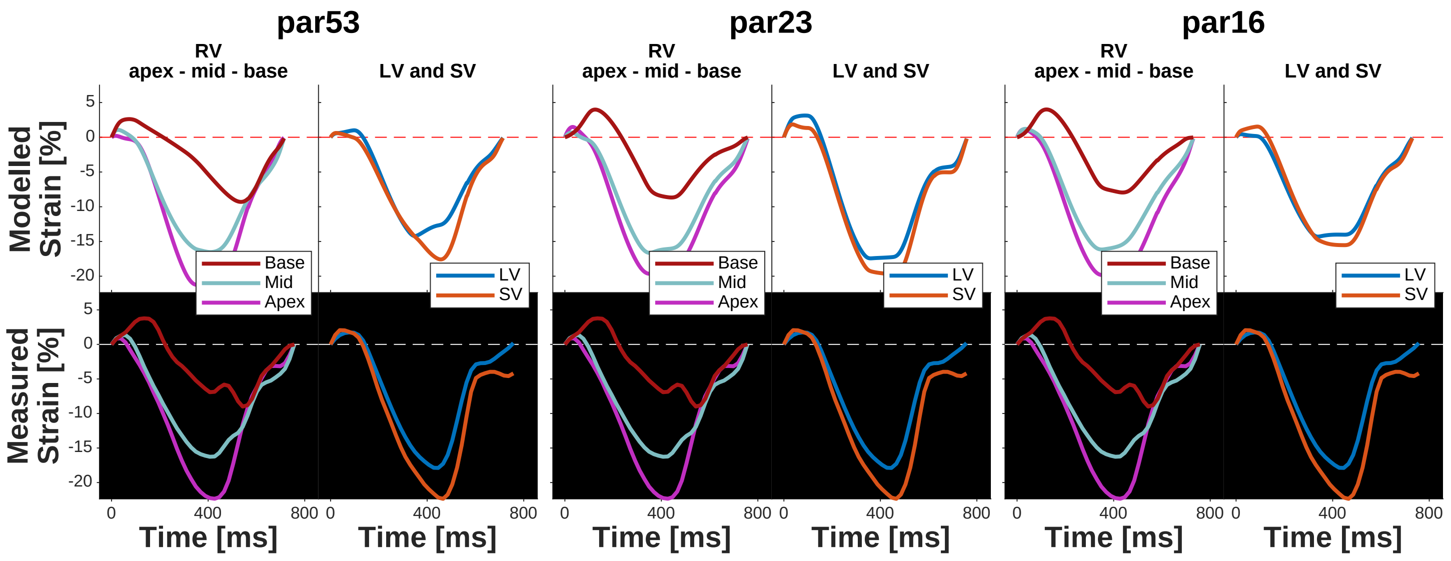

Figure 17: Fits of patient 12

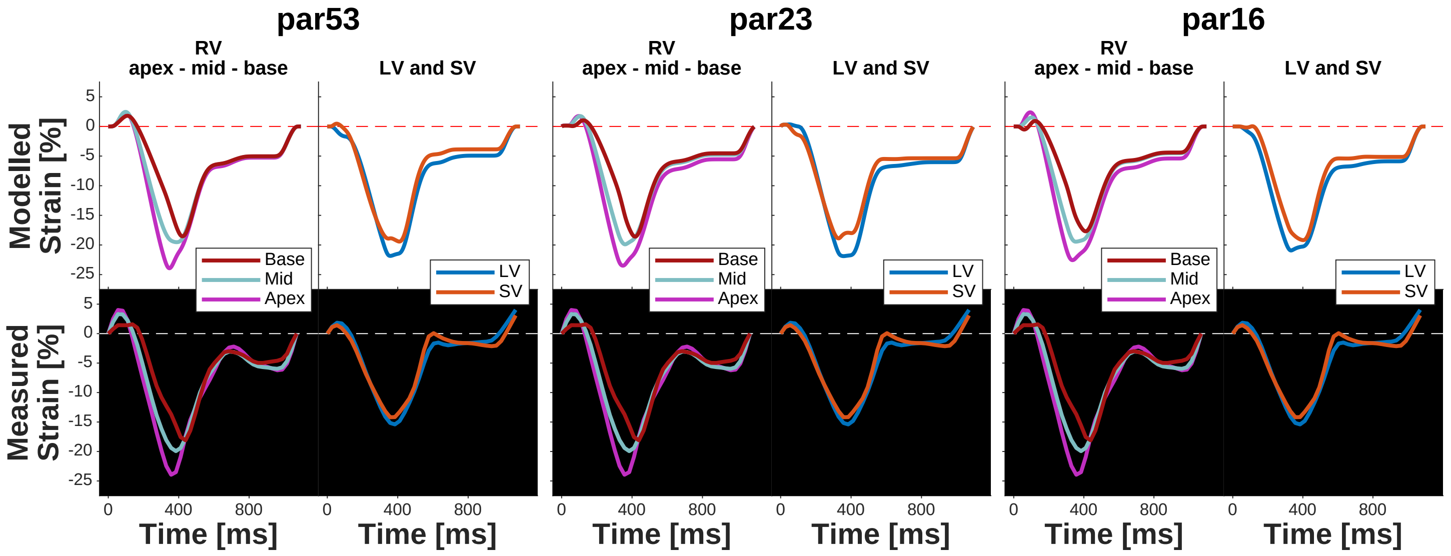

Figure 18: Fits of patient 13

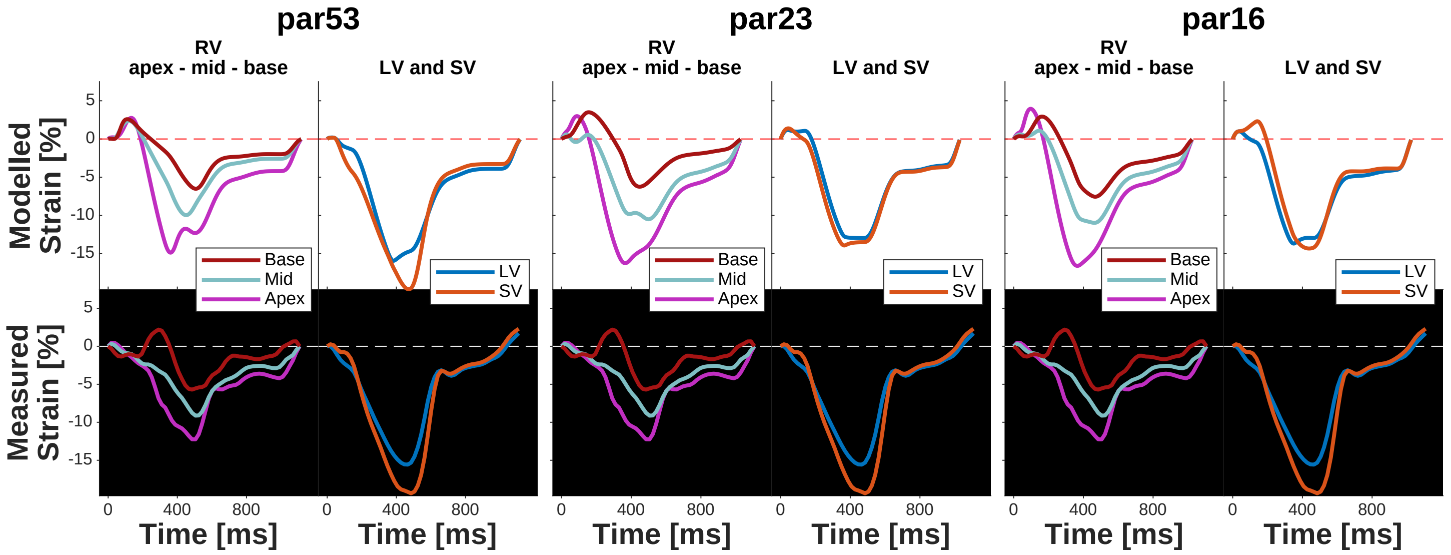

Figure 19: Fits of patient 14

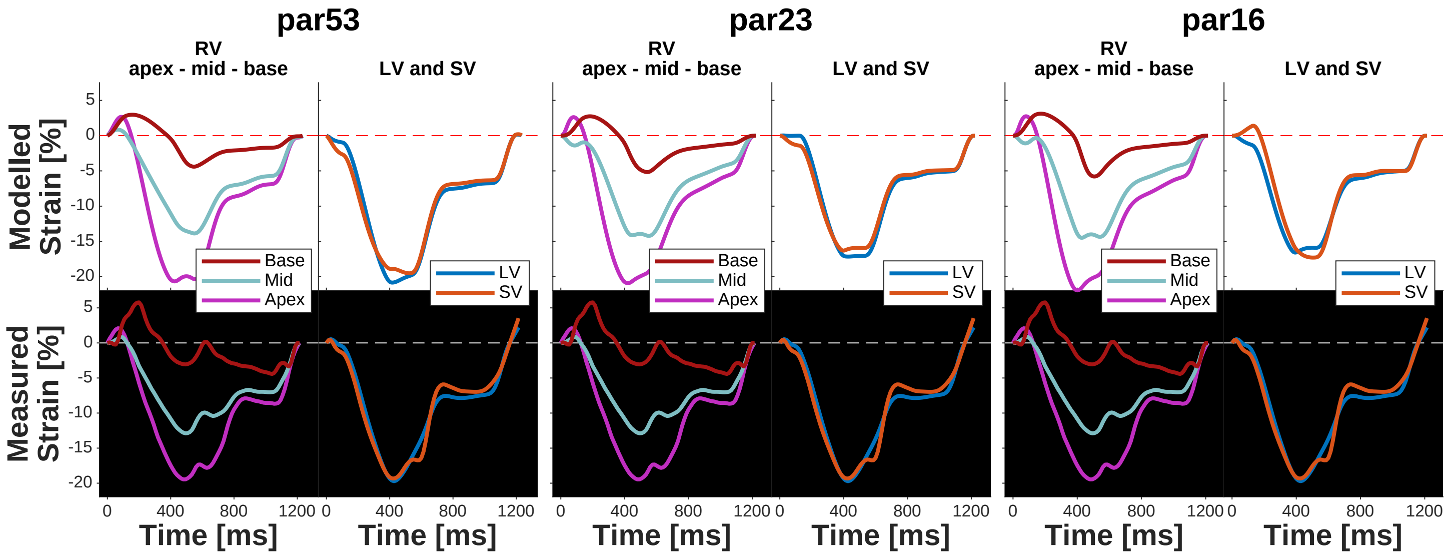

Figure 20: Fits of patient 15
